# Riboflavin kinase and pyridoxine 5′-phosphate oxidase complex formation envisages transient interactions for FMN cofactor delivery

**DOI:** 10.3389/fmolb.2023.1167348

**Published:** 2023-03-28

**Authors:** Maribel Rivero, Sergio Boneta, Nerea Novo, Adrián Velázquez-Campoy, Victor Polo, Milagros Medina

**Affiliations:** ^1^ Departamento de Bioquímica y Biología Molecular y Celular, Facultad de Ciencias, Universidad de Zaragoza, Zaragoza, Spain; ^2^ Instituto de Biocomputación y Física de Sistemas Complejos (BIFI), Universidad de Zaragoza, Zaragoza, Spain; ^3^ Instituto de Investigación Sanitaria Aragón (IIS Aragón), Zaragoza, Spain; ^4^ Centro de Investigación Biomédica en Red en el Área Temática de Enfermedades Hepáticas y Digestivas (CIBERehd), Madrid, Spain; ^5^ Group of Biochemistry, Biophysics and Computational Biology “GBsC” (BIFI, Unizar) Joint Unit to CSIC, Zaragoza, Spain; ^6^ Departamento de Química Física, Universidad de Zaragoza, Zaragoza, Spain

**Keywords:** protein-protein interaction, cofactor delivery, flavin cofactor, riboflavin kinase, molecular modelling, dynamic interaction, pyridoxine (pyridoxamine) 5′-phosphate oxidase

## Abstract

Enzymes catalysing sequential reactions have developed different mechanisms to control the transport and flux of reactants and intermediates along metabolic pathways, which usually involve direct transfer of metabolites from an enzyme to the next one in a cascade reaction. Despite the fact that metabolite or substrate channelling has been widely studied for reactant molecules, such information is seldom available for cofactors in general, and for flavins in particular. Flavin adenine dinucleotide (FAD) and flavin mononucleotide (FMN) act as cofactors in flavoproteins and flavoenzymes involved in a wide range of physiologically relevant processes in all type of organisms. *Homo sapiens* riboflavin kinase (RFK) catalyses the biosynthesis of the flavin mononucleotide cofactor, and might directly interplay with its flavin client apo-proteins prior to the cofactor transfer. Non-etheless, none of such complexes has been characterized at molecular or atomic level so far. Here, we particularly evaluate the interaction of riboflavin kinase with one of its potential FMN clients, pyridoxine-5′-phosphate oxidase (PNPOx). The interaction capacity of both proteins is assessed by using isothermal titration calorimetry, a methodology that allows to determine dissociation constants for interaction in the micromolar range (in agreement with the expected transient nature of the interaction). Moreover, we show that; i) both proteins become thermally stabilized upon mutual interaction, ii) the tightly bound FMN product can be transferred from RFK to the apo-form of PNPOx producing an efficient enzyme, and iii) the presence of the apo-form of PNPOx slightly enhances RFK catalytic efficiency. Finally, we also show a computational study to predict likely RFK-PNPOx binding modes that can envisage coupling between the FMN binding cavities of both proteins for the potential transfer of FMN.

## 1 Introduction

Transient and weak protein-protein and protein-ligand interactions play key roles in innumerable aspects of cellular organization, from the formation of macromolecular complexes to signal transduction. Therefore, their elucidation is crucial to understand the molecular mechanisms of biological processes. Some of these interactions result in a reaction in which the configuration or chemical composition of the interacting molecule is transformed, while others result in no alteration of the interacting molecules (Budayeva and Cristea, 2014). Enzymes catalysing the conversion of reactants usually cooperate in metabolic pathways to obtain energy, precursor metabolites or macromolecules, and have developed chemical and physical mechanisms to control the transport and flux of reactants along the metabolic pathway (Wheeldon et al., 2016). On such occasions, control mechanisms typically consist in metabolite channelling, i.e., direct transfer, from the active site of one enzyme to the active site of the next in a cascade of reactions and transferences. Complexes formed between several consecutive enzymes of a metabolic pathway–known as metabolons–are characterized by transient interactions with physical and functional meanings, where the intermediates are kept within the metabolon by substrate channelling (Zhang and Fernie, 2020; Zhang and Fernie, 2021; Zhang and Fernie, 2022). As a result, metabolites are transferred without (or with minor) releasing into the bulk solution, making the overall process more efficient (Wheeldon et al., 2016; Zala et al., 2017).

Metabolite channelling has been widely studied for reactants, but such information is seldom available in the case of cofactors that, once synthesized, must be transferred and bound to pertinent client apo-proteins to make them functional active holo-proteins. This assemblage may occur directly through a physical interaction between the protein synthesizing the cofactor and the client apo-protein in a sort of cofactor channelling, or in an indirect way, by releasing the newly-synthesized cofactor into the medium where the client apo-protein may take it from. Flavoproteins are a diverse class of proteins, mostly enzymes, that contain flavin mononucleotide (FMN) or flavin adenine dinucleotide (FAD) as cofactors, which enable them to participate in a wide range of relevant processes for the homeostasis of living beings. While some organisms rely heavily on flavin-dependent activities, others maintain them to a minimum (Macheroux et al., 2011; Lienhart et al., 2013; Gudipati et al., 2014; Schall et al., 2020; Eggers et al., 2021; Minjárez-Sáenz et al., 2022). Nonetheless, flavoproteins account for ∼2.5–5% of the proteome of each species, with a clear bypass for the use of FAD over FMN. Most flavoproteins are essential mediators in oxidation-reduction processes, but among the more than 400 different flavin-dependent proteins so far identified ∼10% catalyse non-redox reactions or act as signalling and sensing molecules (Macheroux et al., 2011; Lienhart et al., 2013; Schall et al., 2020). Many flavoenzymes are also suitable biocatalysts due to the selectivity, control and efficiency of the reactions they catalyse (Walsh and Wencewicz, 2013; Hall, 2020), as well as therapeutic targets for the treatment of infectious diseases or mammalian pathological situations where impairments in flavoproteins and flavin homeostasis relate to cancer, cardiovascular diseases, neuromuscular and neurological disorders (Cremades et al., 2009; Sebastián et al., 2018a; Leone et al., 2018; Martinez-Julvez et al., 2018; Leone et al., 2019b; Villanueva et al., 2019; Anoz-Carbonell et al., 2020a; Anoz-Carbonell et al., 2020b; Lans et al., 2020; Novo et al., 2021). A better knowledge of cofactor incorporation into flavoproteins would surely help in the understanding of the metabolic requirements and networks involving them in different organisms, and benefit either industry or health. FMN and FAD are synthesized from the precursor riboflavin (RF) by two consecutive reactions. Firstly, RF is phosphorylated by riboflavin kinase (RFK) in the presence of ATP:Mg^2+^ to form the FMN cofactor, with this conversion being the major rate limiting step in FAD biosynthesis (Karthikeyan et al., 2003; Barile et al., 2016). In a second reaction, FMN:ATP adenylyl transferase (FMNAT) catalyses the adenylylation of FMN to FAD (Torchetti et al., 2011; Barile et al., 2013; Leone et al., 2019a). In human FMNAT there is evidence that the product of its catalysis, FAD, is not released but transferred to some apo-protein in a sort of FAD-chaperoning action where FMNAT binds transitorily to the client apo-protein (Torchetti et al., 2011; Giancaspero et al., 2015). RFK might be expected to also operate as a chaperone in the transfer of FMN to the client apo-protein, as its FMN product is bound tightly and non-covalently and produces allosteric RFK inhibition, moreover being its release the limiting step in overall FMN biosynthesis. Furthermore, this process is regulated by a conformational equilibrium between open (RFK_op_, FMN accessible to the solvent) and closed (RFK_cl_, FMN shielded from the solvent) forms when the products of the reaction are bound (Karthikeyan et al., 2003; Anoz-Carbonell et al., 2020a), suggesting that a client apo-protein accepting the flavin cofactor might be required for FMN release from RFK. The necessity to synchronize cofactor biosynthesis with events of cofactor assembly to client apo-proteins, maintaining thus flavoprotein content and flavin homeostasis, hinders a deep understanding of the molecular basis of the potential FMN-transfer, as well as of its influence in the control of many cellular flavoprotein-dependent processes that depend on FMN supply (Supplementary Table S1) and on its transformation into FAD by FMNAT (Lienhart et al., 2013; Giancaspero et al., 2015).

To address the potential channelling of FMN from RFK to a client apo-protein, here we have chosen as receptor pyridoxine 5′-phosphate oxidase (PNPOx). This is a homodimeric FMN dependent flavoprotein that catalyses the oxidation of pyridoxine 5′-phosphate (PNP) or pyridoxamine 5′-phosphate (PMP) to pyridoxal 5′-phosphate (PLP), with the *in vitro* ability of its FMN-free state (herein, aPNPOx) to be reconstituted to its holo-protein form (di Salvo et al., 2017a; di Salvo et al., 2017b; Musayev et al., 2003). As noted above, the direct transfer of FMN from RFK to aPNPOx should be preceded by interaction between both proteins, although no such complexes have been characterized yet. In this study, we used biophysical binding, kinetics and molecular modelling studies to provide evidence of the interaction between RFK and PNPOx as well as to gain insights into their molecular interaction modes as an initial step in the potential FMN distribution by canalization.

## 2 Materials and methods

### 2.1 Expression and purification of proteins

*Escherichia coli* bacteria containing the pET28a(+) plasmid codifying for PNPOx from *Homo sapiens* were grown at 37°C in Luria Bertani medium containing 30 μg/mL kanamycin and 8 μg/mL of RF. At an O.D._600nm_ = 0.7–0.8, cultures were induced with 1 mM isopropyl-β-D-thiogalactopyranoside (IPTG), incubated overnight at 28°C and then harvested (Musayev et al., 2003). All buffers described below for the purification of PNPOx contained 5 mM 2-mercaptoethanol and 0.2 mM EDTA unless otherwise stated. Cells were resuspended in 50 mM Tris/HCl, pH 8.0, and an EDTA-free protease inhibitor cocktail was added, plus a FMN supplement (10 µM) to ensure the holo-form of the protein. Cells were broken by sonication and soluble protein was collected by centrifugation. Supernatant was incubated with the matrix of a HisTrap HP affinity column (*GE Healthcare*), for 45 min at 4°C and then the column was packed and washed with 20 mM potassium phosphate (KPi), pH 7.8. The protein was eluted with a linear gradient from 0 to 1 M imidazole in 20 mM KPi, pH 7.8 and dialyzed against 20 mM KPi, pH 7.8, 150 mM NaCl and containing 1 mM dithiothreitol (DTT), removing 2-mercaptoethanol and EDTA. PNPOx was further purified by size exclusion chromatography, using a Superdex 200 10/300 GL column (*GE Healthcare*) previously equilibrated with 20 mM KPi, pH 7.8, 150 mM NaCl and 1 mM DTT. Purification and quantification of RFK from *H. sapiens* in its apo-form (herein RFK for RFK-free of products or substrates) was carried out following the procedure previously described (Anoz-Carbonell et al., 2020a). Pure PNPOx and RFK solutions were stored at −80°C in 20 mM KPi, pH 7.8, 150 mM NaCl, 1 mM DTT and in 20 mM PIPES, pH 7.0, respectively, until used.

Protein concentration was quantified by measuring the absorbance at 448 nm for PNPOx (ε_448_ = 16.3 mM^−1^ cm^−1^ per protomer of the homodimer) and at 278 nm for RFK (ε_278_ = 21.43 mM^−1^ cm^−1^) (di Salvo et al., 2017a; Anoz-Carbonell et al., 2020a). Purity was analysed by 12% SDS-PAGE. For PNPOx, the extinction coefficient was experimentally determined at 448 nm by boiling a protein sample for 5 min at 100°C and spinning it to pellet the denatured protein. The released cofactor was then quantitated spectrophotometrically by taking the FMN-free cofactor extinction coefficient value from the literature ( $\varepsilon_{FMN,450}=12.5\;mM^{-1}\;cm^{-1}$ (Aliverti et al., 1999)).

### 2.2 Preparation of the apo-form of PNPOx and holo-form of RFK

PNPOx was purified in its holo-form, but FMN was removed to obtain the apo-form following a procedure described previously with some modifications (Musayev et al., 2003). Briefly, the pH of a ∼10 mg protein solution was lowered to 5.0 by adding 100 mM KPi, pH 5.0, containing 5 mM 2-mercaptoethanol and 0.2 mM EDTA. The solution was then saturated at 20% with ammonium sulphate and loaded onto a Phenyl-Sepharose column (*GE Healthcare*) previously equilibrated with the same buffer. The column was then washed with equilibration buffer until the FMN yellow band was washed out and then the protein was eluted with 0.1 M KPi, pH 7.6, containing 5 mM DTT, 0.2 mM EDTA and 15% propylene glycol. Fractions were collected and their absorbance at 278 nm registered to quantify protein concentration using ε_278_ = 53.36 mM^−1^cm^−1^ (Musayev et al., 2003).

FMN remains strongly bound to RFK and is a potent competitive inhibitor of the RF substrate (*K*_d_ ∼10 nM, *K*_i_^FMN^ 2.5 µM) when it is produced under steady-state by RFK activity, and particularly when it binds to RFK in the presence of ADP:Mg^2+^ (the other reaction product) (Anoz-Carbonell et al., 2020a). Thanks to this, it was possible to stabilize the herein labelled hRFK state (RFK:FMN:ADP:Mg^2+^) in solution. To prepare it, RFK was loaded with the products of its reaction, FMN and ADP, in buffer containing 0.3 mM MgCl_2_. Both FMN and ADP were added in a 1:3 ratio and their excess was removed by centrifugation, being the final ratio ∼1:1.

### 2.3 Isothermal titration calorimetry (ITC)

ITC measurements were performed using an Auto-iTC200 MicroCal calorimeter (*Malvern-Panalytical*) thermostated by following previously established procedures (Anoz-Carbonell et al., 2020a). For regular experiments, the sample cell contained either aPNPOx or PNPOx (5 μM considering the protein a homodimer) and was titrated with RFK-free or hRFK samples (50–100 µM). Measurements were carried out at 25°C and the buffer dissolving both the ligand and the receptor was either 20 mM KPi, pH 7.8, or 20 mM PIPES, 0.3 mM MgCl_2_, pH 7.0. All solutions were degassed at 15°C for 1 min beforehand. The association constant (*K*_a_), the enthalpy change (Δ*H*) and the binding stoichiometry (*N*), as well as the cooperativity constant (α) and cooperativity enthalpy (Δ*h*) when appropriate, were estimated through non-linear least-squares regression of the experimental data employing either single-ligand or two-ligand binding site models implemented in Origin 7.0 (OriginLab). The dissociation constant (*K*_d_ ), entropic contribution (−TΔ*S*) and Gibbs free energy (Δ*G*) of the binding reaction were obtained from basic thermodynamic relationships, ΔG = −RTLnK_a_, ΔG = ΔH–TΔS and K_d_ = 1/K_a_, where *R* is the ideal gas constant and T is the absolute temperature in K.

### 2.4 Thermal denaturation assays

Thermal denaturation curves were followed by changes in fluorescence emission of the protein aromatic residues upon unfolding (excitation at 280 nm and emission collection at 330 nm) and in the FMN flavin cofactor fluorescence emission upon its release from the protein (excitation at 450 nm and emission at 530 nm). Denaturation curves were recorded from 15°C to 90°C with scan rates of 1.5°C/min using a Cary Eclipse Fluorescence Spectrometer (*Agilent*). Samples contained RFK and/or homodimeric PNPOx at 2 and 1 µM respectively (either when free or in mixtures). Curves were roughly normalized to values between 0 and 1 and, unless otherwise stated, the individual experimental data sets were globally analysed as one-transition (i.e., two-step process, native ↔ unfolded, N ↔ U) or two-transition process (i.e., three-state process, native ↔ intermediate ↔ unfolded, N ↔ I ↔ U) by applying the following equations (Villanueva et al., 2019):

$$S_{obs}=\frac{S_{N}+m_{N}T+\left(S_{U}+m_{U}T\right)e^{-\left(\Delta G/RT\right)}}{1+e^{-\left(\Delta G/RT\right)}}$$ (1)

$$S_{obs}=\frac{S_{N}+m_{N}T+\left(S_{I}+m_{I}T\right)\;e^{-\left(\Delta G_{1}/RT\right)}+\left(S_{U}+m_{U}T\right)e^{-\left(\left(\Delta G_{1}+\Delta G_{2}\right)/RT\right)}}{1+e^{-\left(\Delta G_{1}/RT\right)}+e^{-\left(\left(\Delta G_{1}+\Delta G_{2}\right)/RT\right)}}$$ (2)

where *S*_obs_ is the measured protein signal at a given temperature (*T*), *S*_N_, *S*_I_, and *S*_U_ are the intrinsic signals (y-axis intercept) of the native, intermediate and unfolded protein conformations at 0 K, respectively and *m*_N_, *m*_I_, and *m*_U_ are the slopes of the linear temperature dependence of those signals, respectively. The free energy differences are temperature-dependent according to: $\Delta G_{i}=\Delta H_{i}\left(1-\frac{1}{T_{mi}}\right)+\Delta C_{Pi}\left(T-T_{mi}-T\ln\frac{T}{T_{mi}}\right)$ , where Δ*H*_i_, *T*_mi_, and Δ*C*_Pi_ are respectively the van’t Hoff enthalpy, midtransition temperature and heat capacity change for each unfolding transition, and *R* is the ideal gas constant. Δ*C*_Pi_ was fixed to expected values along the fitting process (since it often shows considerable degeneracy and large uncertainty), and theoretical unfolding enthalpies for RFK and aPNPOx were evaluated by scaling with the number of residues according to ΔH at 60°C = 700 × Ncal/mol (Robertson and Murphy, 1997).

### 2.5 Fast kinetics measurements to evaluate FMN binding to PNPOx

Measurements to evaluate pre-steady state rates for FMN binding to aPNPOx were registered using stopped-flow spectroscopy on an Applied Photophysics SX17. MV spectrophotometer, using the ProData SX software (*Applied Photophysics Ltd.*) for acquisition of changes in flavin fluorescence and kinetic data analysis. Fast kinetic measurements were carried out at 25°C in PIPES 20 mM, pH 7.0, 0.3 mM MgCl_2_. In short, 0.1 μM of homodimeric aPNPOx was mixed with equal volumes of either FMN or hRFK samples at ratios 1:2, 1:4, 1:8, and 1:16, where the indicated concentrations are the final ones in the stopped-flow observation cell. Evolution of flavin fluorescence after mixing was measured with an excitation wavelength of 445 nm, while fluorescence emission was recovered using a >530 nm cut-off filter. At least three reproducible traces acquired at each assayed time and concentration were used to determine mean kinetic parameters by their fitting to exponential functions, according to: $y=C+\underset{i}{\sum }A_{i}e^{-k_{obs,i}t}$ , where *A*_i_ and *k*_obs,i_ are the amplitude of the fluorescence change and the observed kinetic constant for a particular spectroscopic process i that contributes to the overall time dependent fluorescence change and C is the fluorescence limiting value. *k*_obs_ values showing a linear increasing dependence on the flavin concentration were fitted to a one-step model associated to the binding equilibrium of the flavin ligand to aPNPOx, whose kinetics can be represented by the following equation *k*_obs_ = *k*_on_ ⋅ [FLV]+*k*_off_, where *k*_on_ and *k*_off_ are the kinetic rate constants for complex formation and dissociation, respectively, and FLV will stand for any type of flavin source (namely, FMN, RF, hRFK, *etc.*). *k*_obs_ values showing a hyperbolic dependence on the FLV concentration were fitted to a potential induced fit model that would allow to estimate apparent parameters for protein-protein binding (*K*_*d*_) as well as for rates of conformational reorganizations to achieve the final complex state (*k*_*r*_):

$$k_{obs}=k_{r}.\frac{\left[FLV\right]}{K_{d}+\left[FLV\right]}$$ (3)

### 2.6 Kinetic assays to evaluate catalytic PNPOx and RFK activities

PNPOx activity was assayed at 37°C in 50 mM Tris/HCl, pH 7.6, containing 0.3 mM MgCl_2_ and 1 mM DTT, added just before the assays were performed. Kinetics measurements were performed in a reaction mixture containing 0.1 µM of either aPNPOx or PNPOx homodimers, varying PNP substrate concentration (0–20 µM) and, in the case of aPNPOx, keeping constant concentrations of the FMN cofactor provided by either RFK in presence of its reaction substrates RF and ATP:Mg^2+^ (herein sRFK), hRFK, or FMN itself. In all assays the reaction was initiated with the addition of PNP. Kinetic measurements for aPNPOx activity were also carried out in reaction mixtures containing a PNP constant concentration (18 µM) while varying the FMN donor concentration (0.025–2 µM) and incubation time. For incubation times equal to 0 min, the reaction was initiated by adding the FMN donor (FLV), while for incubation times of 5 min the reaction was initiated with PNP. In all cases, the progress of the reaction was studied using a Cary 3500 UV-Vis Spectrophotometer (*Agilent*) and followed at 414 nm, where the characteristic aldimine PLP-Tris product absorbs maximally (ε_414_ = 5.9 mM^−1^ cm^−1^) (Musayev et al., 2003), and initial velocities were determined over the first 2 min of reaction.

Stopped-flow spectrophotometry (*Applied Photophysics Ltd.*) was also used to evaluate kinetic traces from the starting mixing point for the production of PLP by aPNPOx samples getting activated by FMN from different sources. In these assays, aPNPOx was simultaneously mixed with its PNP substrate plus different sources of FMN (either free FMN, sRFK or hRFK) to convert the inactive aPNPOx to the active PNPOx form. For sRFK samples two different reactions mixtures were prepared. One contained in syringe 1 RFK together with aPNPOx while substrates of both enzymes were placed in syringe 2 (equivalent to sRFK). In the other assay, aPNPOx was alone in syringe 1 and RFK was placed in syringe 2 together with its substrates and PNP (denominated sRFK_2_). Final concentrations in the stopped-flow mixture chamber were [PNPOx homodimer] = 0.05 µM, [PNP] = 25 μM, [FLV] = 0.25 µM. Measurements were performed in 50 mM Tris/HCl pH 7.6, 0.3 mM MgCl_2_, 1 mM DTT at 25°C. Spectral evolution was recorded in the 300–800 range using a photodiode array detector (*Applied Photophysics Ltd.*) and formation of the product, aldimine PLP-Tris, was determined by absorbance changes at 414 nm. All experiments were run in at least triplicate.

The influence of the presence of aPNPOx in the RFK activity was assessed at 25°C in 500 µL of 20 mM PIPES, pH 7.0, 0.3 mM MgCl_2_ and 1 mM DTT. Different concentrations of aPNPOx homodimer (0–50 nM) were mixed with variable concentrations of RF (0.6–40 µM) and a saturating concentration of ATP (400 µM). In all cases, reactions were initiated by addition 40 nM RFK to reaction mixtures pre-incubated at 25°C, being then incubated at the same temperature for 1 min. Then, reactions were stopped by boiling the samples at 100°C for 5 min. Flavin composition of the supernatant was resolved by an Alliance HPLC system (*Waters*) equipped with a 2,707 autosampler and HSST3 column (4.6 × 50 mm, 3.5 mm; Waters) preceded by a pre-column of the same material (4.6 × 20 mm, 3.5 mm, Waters). An aliquot of 15 µL of each mixture was applied and the chromatography was developed at 1 mL/min with a 6 min isocratic program of methanol 40% (vol/vol) in 5 mM ammonium acetate pH 6.0 (Serrano et al., 2013). FMN concentrations were quantified using a standard curve acquired in the same conditions. The observed steady-state rates for FMN production (v_0_) were determined in unit of µmoles of flavin produced per second per µmol of enzyme (v_0_/[e]). All experiments were performed in triplicate.

### 2.7 Building of structural models for potential interacting complexes

Energetically optimized models for the assembly of *H. sapiens* RFK and PNPOx were built by subsequent steps of molecular dynamics (MD) and protein-protein docking simulations. Structural models representing different ligation (apo- and holo-protein), conformational (open or closed) and/or oligomeric (monomer or homodimer) states for the evaluated proteins were used. A PNPOx crystallographic structure including FMN and PLP was used to build starting models (PDB: 1NRG) in either its monomer or homodimer states, and corresponding aPNPOx models were built by removing FMN and PLP. For RFK, structures were those in complex with both reaction products, ADP:Mg^2+^ and FMN, in its open (PDB: 1P4M, hRFK_op_) and closed (PDB: 1Q9S, hRFK_cl_) conformations.

For MD simulations, structural models were first prepared by removing all water molecules and then protonated to pH 7.0 by using the PROPKA software (Olsson et al., 2011). All-atom MD simulations were performed using GROMACS 2018.4 (Abraham et al., 2015) and the AMBER ff03 force field to parametrize the protein (Duan et al., 2003). Topologies for standard residues were assigned using *pdb2gmx* from the GROMACS toolkit, while FMN and ADP were parametrized using *ab initio* methods. Partial charges for each atom were assigned by the restrained electrostatic potential (RESP) method using Multiwfn (Lu and Chen, 2012) after a structure optimization at HF/6-31G(d,p) level in Gaussian 09 (Frisch et al., 2016). These charges were employed as starting point for the parametrization with GAFF (Wang et al., 2006) force field by *antechamber* (Wang et al., 2004) through ACPYPE (Sousa da Silva and Vranken, 2012). Periodic boundary conditions were set by placing the system in the centre of a rhombic dodecahedron box, solvated with explicit water molecules modelled by TIP3P. Every system was neutralized by replacing random solute molecules with sodium ions. When required, before neutralization, the Mg^2+^ atom was added as an extra ion, and placed at the protein binding site. A steepest descent minimization was performed to avoid close contacts or clashes and the resulting systems were used as starting structures to perform MD simulations. Before running the production and data acquisition, desired conditions were achieved with sort simulations of 500 ps with an NVT ensemble, and generating random initial velocities according to a Boltzmann distribution for 310 K, and a successive 500 ps simulation with an NPT ensemble and 1 atm. In both equilibration steps, the movement of the atoms of proteins and ligands were restrained with a 1,000 kJ/(mol·nm) harmonic potential. Once desired conditions were achieved, the productive MD phase was run with unrestrained positions (except for bonds including hydrogen atoms, which were restrained by applying LINCS algorithm (Hess, 2008)) and an NPT ensemble at 310 K. *Leap-frog* integrator with 1 fs time steps was used, collecting data every 10 ps. Particle Mesh Ewald method for long range electrostatic interactions, Parrinello-Rahman method for pressure control and modified Berendsen method for temperature equilibration were used. Simulation times of up to 300 ns were reached and five replicas were performed for each trajectory. All MD simulations were run on CIERZO supercomputer system at BIFI.

Dockings between RFK and PNPOx states were evaluated by using the above mentioned crystallographic structures of these proteins and the pyDockWeb server (Jiménez-García et al., 2013). This is a rigid-body docking method that provides the best docking orientations scored on the basis of shape complementary, together with electrostatics and desolvation binding energies. For each docking, the 100 lowest energy docking poses were generated as PDB files, used to calculate the Normalized interface propensity (NIP) values for each residue in the coupling and checked to identify the most favourable conformations for FMN transference. To identify further suitable orientations for FMN transference additional dockings were performed using one energetically optimized RFK_cl_ conformation and aPNPOx, and evaluated including restrains that included some of the following as involved in the interaction: namely, F23, R25, T51, W74, P76-K79, N80 or N115-D117 for RFK and Q71, P73, D74, E77, K100 or S160 chain A and/or Q222-R225 chain B for PNPOx (Chelliah et al., 2006). Since pyDockWeb does not consider ligands, docking models were in apo-forms regardless of whether they were included in the starting structures. Then, in selected docked structures RFK was substituted by different equilibrated hRFK_op/cl_ models above obtained after MD simulations. Finally, the obtained hRFK_op/cl_-aPNPOx complexes were minimized and relaxed by MD simulations to energetically optimize and explore their conformational space, following the protocol described above. PyDockWeb resources (Jiménez-García et al., 2013), PyMOL (Delano, 2002), VMD (Humphrey et al., 1996) and/or GROMACS package tools (Abraham et al., 2015) were used to evaluate resulting docking poses as well as MD trajectories. Electrostatic surface potentials (ESP) of each variant were calculated at pH 7.0 using the APBS-PDB2PQR server (https://www.poissonboltzmann.org/) and then plotted using PyMOL.

### 2.8 General data analysis

PyMOL (Delano, 2002) and the graphic software Origin (OriginLab, United States) were used for data analysis and figure production.

## 3 Results

### 3.1 Isothermal titration calorimetry, thermal stability and rates for flavin binding assays support physical interaction between RFK and PNPOx

**FIGURE 1:**
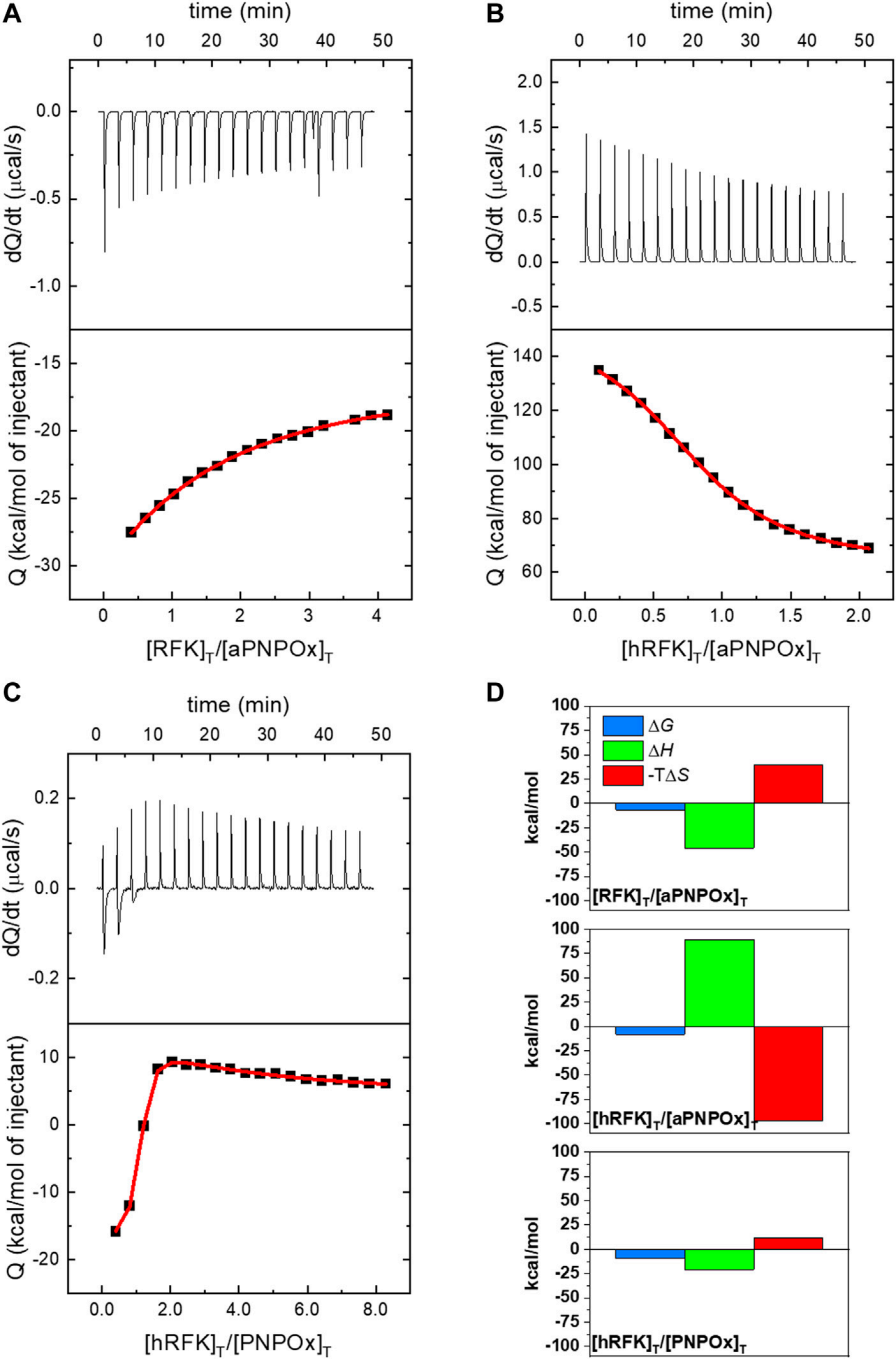
Isothermal calorimetric titrations for the interaction of PNPOx and RFK. ITC profiles at 25°C for the titration of **(A)** aPNPOx with RFK, **(B)** aPNPOx with hRFK and **(C)** PNPOx with hRFK. The upper panels show the thermograms for the interactions, whereas the bottom panels show the corresponding binding isotherms with integrated heats. **(A)** and **(B)** show measurements performed in 20 mM potassium phosphate (KPi), pH 7.8, while measurement in **(C)** was performed in 20 mM PIPES, 0.3 mM MgCl_2_, pH 7.0. Data were fitted to home-derived models implemented in Origin 7.0 for a single binding site in **(A)** and **(B)** and for two identical binding sites with cooperativity in **(C)** (continuous red lines in binding isotherms). **(D)** Thermodynamic contributions to the binding as derived from panels **(A)**, **(B)**, and **(C)** with Gibbs energy (Δ*G*), enthalpy (Δ*H*) and entropic contribution (−TΔ*S*) represented in blue, green and red bars, respectively. In **(C)** contributions are only shown for the first binding site. Data correspond to the buffers in the other panels, so **(C)** data are not comparable directly with **(A)** and **(B)**.

ITC was used to assess the ability of RFK, either cofactor-free or in the hRFK form, to associate to PNPOx. Thermograms shown in Figures 1A, B, obtained in 20 mM KPi, pH 7.8, clearly indicated protein-protein binding. Interaction between both proteins was further confirmed by titrations at different temperatures, exchanging receptor and titrating ligands in the experimental setup (i.e., reverse titrations) or using 20 mM PIPES, 0.3 mM MgCl_2_, pH 7.0 as alternative buffer (Figure 1C), to ensure a good outcome from experiments using different samples. Because the KPi has very low ionization enthalpy, thermograms in this buffer allowed to determine intrinsic thermodynamic parameters, such as the change in enthalpy (Δ*H*) and entropic contributions to the binding. Noticeably, these data provided an endothermic binding isotherm for the aPNPOx:hRFK coupling, while the aPNPOx:RFK interaction was exothermic. In both cases, binding stoichiometry was close to 1 (N respectively 0.86 and 1.1), suggesting that the aPNPOx homodimer is the functional unit and that it binds one molecule of either hRFK or RFK. Apparent *K*_d_ values for these interactions were respectively 0.9 ± 0.1 and 14 ± 1 μM, and the corresponding thermodynamic parameters, Δ*H*, Δ*G,* and –TΔ*S*, were 89 ± 1, −8.2 ± 0.3, and −97 ± 2 kcal/mol for the aPNPOx:hRFK interaction and −46 ± 0.1, −6.6 ± 0.3, and 39.4 ± 0.3 kcal/mol for the aPNPOx:RFK one (Figure 1D). These parameters confirm large entropic contributions driving the interaction of hRFK with aPNPOx and enthalpic contributions favouring that of RFK, as well as stronger affinity for the binding of hRFK. In addition, data for the titration of the PNPOx with hRFK (Figure 1C) in PIPES also indicated two binding sites on the oxidase homodimer, but in this case binding of the two hRFK molecules showed negative cooperativity. PNPOx is a homodimer in solution and, consequently, may bind two hRFK molecules, one in each protomer. Due to symmetry, the two sites are identical and, in principle, they may bind hRFK in an independent or cooperative fashion. Because of the biphasic binding isotherm observed in the calorimetric titration, the two sites must behave in a cooperative fashion, and the binding model accounts for that possibility through a cooperativity interaction constant α together with the intrinsic association constant *K*_a_. The parameter α quantifies the cooperative phenomenon in the binding (first hRFK molecule binds with affinity *K*_a_, and the second hRFK molecule would bind with affinity *K*_a_α), implicitly considering statistical factors (e.g., lower available number of binding sites for the second binding molecule compared to the first binding molecule) and considering any quantitative deviation from the non-cooperative binding scenario. For example, the fractional population of the singly-liganded complex ([ML]/[M]_T_) at the mid-equivalence point (half overall binding saturation fraction) is a function of the cooperativity parameter: 1/(1 + sqrt(α)), which is 0.5 for binding independence (α = 1), less than 0.5 for positive cooperativity (α > 1), and more than 0.5 for negative cooperativity (α < 1) (Freire et al., 2009; Vega et al., 2015). The following parameters were derived for the interaction at the first binding site: *N* = 0.96, *K*_d_ 0.13 ± 0.01 µM, Δ*H* −21 ± 0.3 kcal/mol, Δ*G* −9.3 ± 0.3 kcal/mol, and –TΔ*S* 11.7 kcal/mol, being α and cooperativity enthalpy (Δh) for the second biding site respectively 0.015 and 87 kcal/mol. Such type of cooperative behaviour has already been described in other homodimeric flavoenzymes for the binding of flavin cofactors at the protein-protein interface of both protomers of the dimer (Clavería-Gimeno et al., 2017). In general, *K*_d_ values in the low micromolar range indicate that the resulting transient complexes are more organized than the isolated proteins. Nonetheless, the higher *K*_d_ value determined for the aPNPOx:RFK interaction and the negative cooperativity for the PNPOx:hRFK interaction envisage differential and specific binding modes dependent on the RFK and PNPOx ligation states.

**FIGURE 2:**
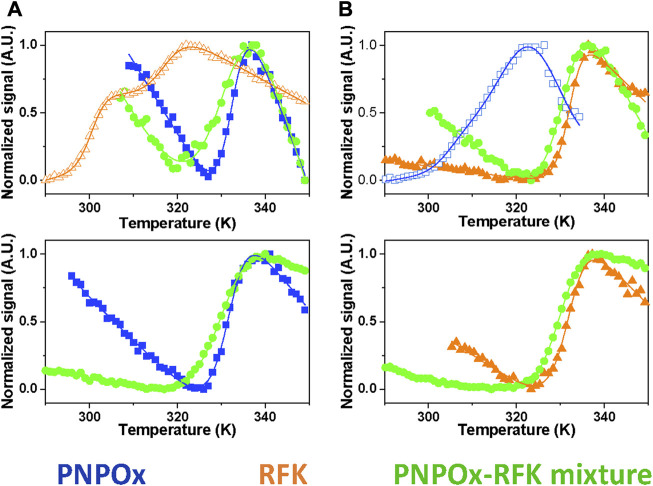
Impact of the potential RFK-PNPOx interaction on thermal stability of the individual proteins. Thermal unfolding curves for samples of **(A)** PNPOx, RFK and a PNPOx-RFK mixture, and **(B)** hRFK, aPNPOx and a hRFK-aPNPOx mixture. The upper and bottom panels respectively monitor changes in tryptophan and FMN fluorescence. Data for PNPOx, RFK and their mixtures are shown respectively in blue squares, orange triangles and green circles, with closed and open symbols for single proteins respectively denoting their holo- and apo-forms. The curves are shown roughly normalized from 0 to 1, and their global fits to one-transition unfolding models are represented by the continuous lines, with the only exception of RFK in panel **(A)** that best fits to a two-transitions model. Curves were recorded in 20 mM PIPES pH 7.0.

The ability of PNPOx to interact with RFK was further explored by thermal unfolding curves recorded for the single proteins in different ligation states as well as when mixed, allowing to evaluate whether their individual stability is different from that of their potential complexes. Unfolding parameters matched in general when obtained by following changes in the intrinsic tryptophan fluorescence (upon increasing solvent exposition) and by increase in flavin fluorescence (upon its release from the protein) (Figure 2). RFK unfolding was best described by a three-state equilibrium unfolding process, while two-states unfolding processes were observed for the remaining analysed samples (Figure 2; Table 1). Both proteins were considerably less stable in apo-forms regarding their respective holo-forms, demonstrating an increase of T_m_ for hRFK and PNPOx of ∼ 32°C and ∼ 24°C, respectively. Noticeably, mixed samples containing one of the proteins in the apo-form and the other one in the holo-form evidenced denaturation at T_m_ values closer to those of the holo-forms, suggesting the presence of the partner holo-protein provides protection of the apo-forms against thermal denaturation. Moreover, T_m_ values for these mixtures envisaged a slight destabilization of the holo-protein form. Regarding unfolding enthalpies, the first of the two experimentally determined values for RFK (Table 1) fitted well with the theoretical (110 kcal/mol at 60°C), pointing to the second process related to some aggregation or precipitation. On the contrary, aPNPOx unfolding enthalpy was much lower than the theoretically estimated (180 kcal/mol at 60°C), while being associated with an apparent broad single-transition trace with a small slope that reflected low unfolding cooperativity. This may indicate the existence of at least two indistinguishable transitions, which may reflect dissociation and unfolding of each protomer. Altogether, the shifts in stability and unfolding enthalpies when proteins were mixed further constituted indirect evidence of the interaction between them.

**TABLE 1:** Influence of RFK and PNPOx ligation states and mutual interplay in their thermal stability parameters. Values were obtained by fitting fluorescence thermal denaturation curves, followed by changes in tryptophan and flavin (when applicable) fluorescence to two species unfolding mechanisms, with the only exception of RFK that followed a three species unfolding mechanism. Data were obtained in 20 mM PIPES pH 7.0 from 283.15 to 363.15 K (*n* ≥ 2, mean ± SD).

Sample	Fluorescent probe	T_m₁_ (K)	T_m₂_ (K)	ΔH_₁_ (Kcal/mol)	ΔH_₂_ (Kcal/mol)
RFK	Trp	300 ± 1	317 ± 1	100 ± 11	73 ± 12
aPNPOx	Trp	313 ± 2		53 ± 5	
hRFK	FMN	332 ± 1		97 ± 6	
	Trp	332 ± 1		134 ± 7	
PNPOx	FMN	331 ± 1		95 ± 3	
	Trp	333 ± 1		119 ± 7	
PNPOx/RFK	FMN	330 ± 1		63 ± 1	
	Trp	330 ± 1		53 ± 5	
aPNPOx/hRFK	FMN	330 ± 1		78 ± 2	
	Trp	331 ± 1		83 ± 3	
aPNPOx/RFK	Trp	316 ± 1		62 ± 8	

**FIGURE 3:**
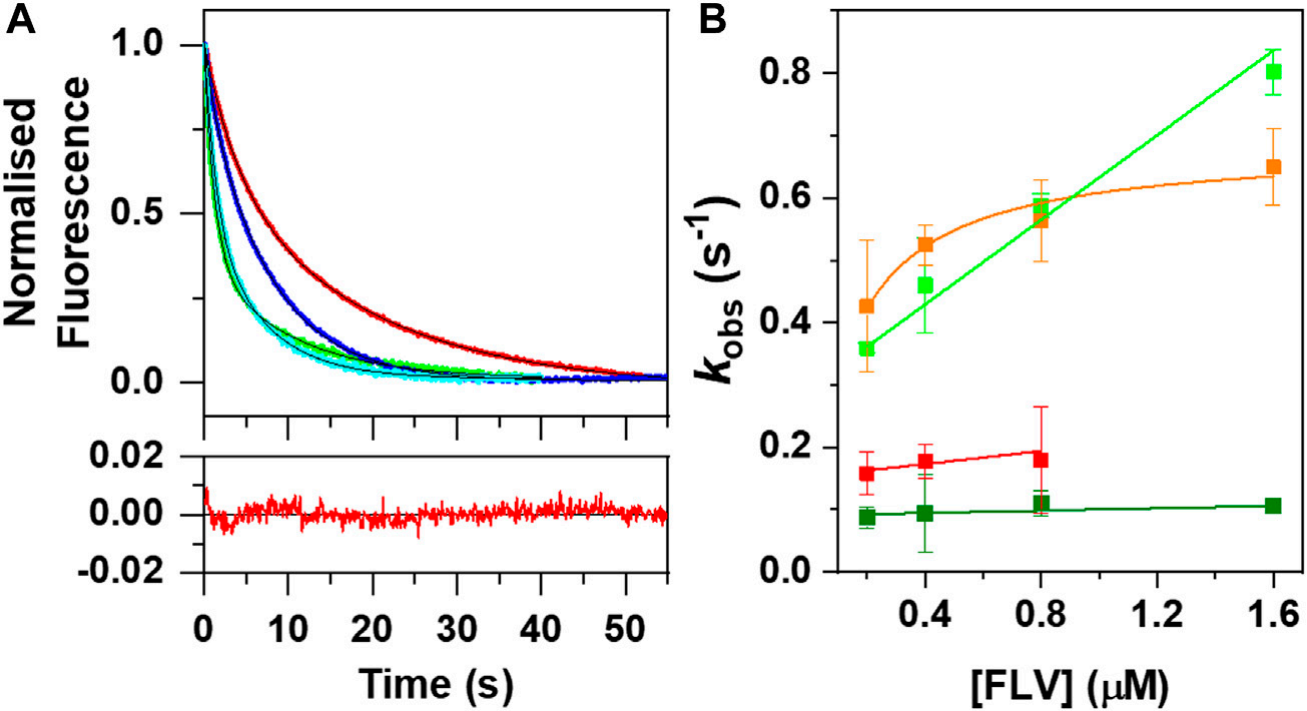
Kinetics of FMN fluorescent changes upon binding to aPNPOx when taken from either the bulk solution or hRFK. **(A)** Normalized evolution of kinetic changes in fluorescence upon mixing aPNPOx (0.1 μM, concentration consider as homodimer) with either FMN or hRFK at equimolecular flavin concentrations (0.2 μM) and in an 8-fold excess of the flavin (1.6 µΜ). Data for equimolecular and 8-fold flavin concentrations are respectively shown in red and green for FMN and in blue and cyan for hRFK (herein FLV, as FMN and hRFK), while fits to a two exponential process are shown in black. The bottom panel shows the residuals for the fitting of the data for flavin binding to aPNPOx when provided by FMN at equimolecular FLV concentration. **(B)** Evolution of *k*_obs1_ and *k*_obs2_ as a function of FLV concentrations. *k*_obs1_ and *k*_obs2_ values are shown respectively in light and dark green for FMN, and in orange and red for hRFK. Measurements were carried out in a stopped-flow equipment in 20 mM PIPES pH 7.0 at 25°C. *k*_obs_ showing linear increasing dependences were fitted to the binding equilibrium of the flavin ligand to aPNPOx according to *k*_obs_ = *k*_on_ ⋅ [FLV]+*k*_off_, while *k*_obs_ showing hyperbolic dependences were fitted to a potential induced fit model according to Eq. 3.

Fast kinetic changes in flavin fluorescence yields upon mixing with RFK have been shown to be a good method to evaluate flavin internalization rates by stopped-flow spectrophotometry (Sebastián et al., 2017; Sebastián et al., 2018b; Anoz-Carbonell et al., 2020a). Here, we have exploited this methodology to evaluate FMN incorporation into aPNPOx. Fast mixing of aPNPOx with FMN resulted in shielding of cofactor fluorescence, in agreement with its environment becoming less polar upon entrapment into PNPOx (Figure 3A). FMN fluorescence decays were best fitted to a bi-exponential mechanism at all assayed FMN concentrations, indicating that FMN entrapment within the protein might occur through a two steps process and an intermediate conformation. The amplitude of the slower process (*A*_*2*_) dominated the fluorescence shielding at FMN concentrations equal to or double the number of FMN binding sites *per* aPNPOx protomer, becoming *A*_*1*_ and *A*_*2*_ similar when flavin concentration further increased. Rates observed for these two detected processes, *k*_obs1_^FMN^ and *k*_obs2_^FMN^, linearly increased with FMN concentrations (Figure 3B), allowing to figure out apparent kinetic rate constants for FMN association and dissociation to the protein along these two consecutive shielding processes. The calculated values for *k*_on1_^FMN^, *k*_on2_^FMN^, *k*_off1_^FMN^ and *k*_off2_^FMN^ (respectively 0.34 ± 0.02 s^−1^ μM^−1^, 0.009 ± 0.005 s^−1^ μM^−1^, 0.29 ± 0.01 s^−1^, and 0.09 ± 0.01 s^−1^) are indicative of the first shielding process being: i) considerably faster than the second and ii) the one contributing stronger to FMN binding. Interestingly, kinetic traces for FMN incorporation into aPNPOx from hRFK followed overall bi-exponential flavin fluorescence decays similar to those of free FMN. Moreover, increase in flavin fluorescence indicative of FMN release to the bulk before transferred to aPNPOx was not detected. Nonetheless, *k*_obs1_^hRFK^ values showed a saturation profile on hRFK concentration (Figure 3B) and resulted faster than those of *k*_obs1_^FMN^ at the lower concentrations assayed. Such different behaviour confirmed that, from the kinetics point of view, the incorporation of FMN into aPNPOx from hRFK was modulated by the interplay between both proteins and might follow a hRFK+aPNPOx⇆hRFK:aPNPOx→RFK:PNPOx process characterized by and an apparent dissociation constant in the low micromolar range (∼1 µM) and a limiting rearrangement constant of 0.7 ± 0.01 s^−1^. *k*_obs2_^hRFK^ values linearly increased with flavin donor concentration and were slightly larger than *k*_obs2_^FMN^. Nonetheless, they derived in *k*_on2_^hRFK^ and *k*_off2_^hRFK^ of 0.05 ± 0.03 s^−1^ μM^−1^ and 0.05 ± 0.02 s^−1^, respectively, only marginally above of those for free FMN.

### 3.2 PNPOx and RFK influence each other in catalysis and ligand binding steps

**FIGURE 4:**
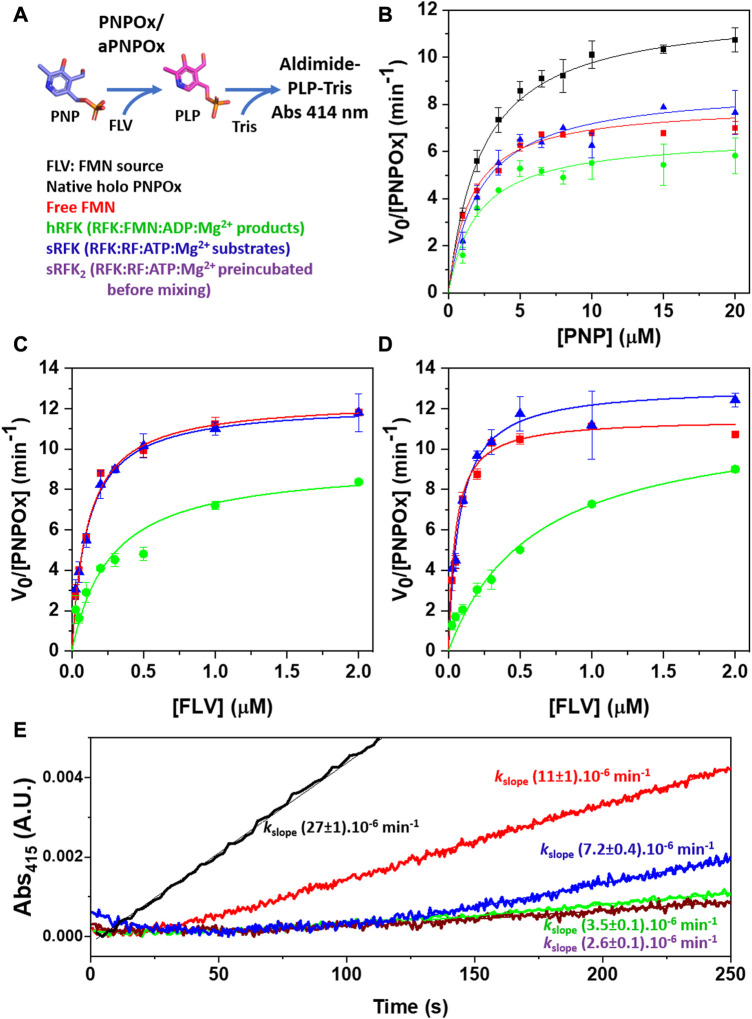
Influence of FMN source in the recovery of the aPNPOx activity to transform PNP into PLP. **(A)** Scheme of the PNPOx steady-state enzymatic assay: upon PNP transformation into the PLP product, PLP accumulation is detected by formation of the aldimine PLP-Tris adduct that absorbs maximally at 414 nm. **(B)** Michaelis-Menten plots for the formation of the PLP-Tris adduct upon PNP transformation by PNPOx, as well as by aPNPOx reconstituted by free FMN, by hRFK (RFK pre-incubated with the products of its reaction, RFK:FMN:ADP:Mg^2+^) and by a just prepared mixture of RFK with its RF and ATP:Mg^2+^ substrates (RFK:RF + ATP:Mg^2+^, sRFK). Dependence of aPNPOx activation on FMN concentration when measuring PLP-adduct formation **(C)** upon mixing and **(D)** after 5 min pre-incubation with the FMN source while keeping PNP substrate at saturating concentrations. All measurements were performed in 50 mM Tris/HCl pH 7.6, 0.3 mM MgCl_2_, 1 mM DTT at 37°C. **(E)** Fast kinetics evolution for the formation of the PLP-Tris adduct as followed in the stopped-flow equipment upon the mixing of PNPOx (syringe 1) with PNP (syringe 2). In all cases syringe 2 also contained the source of FMN (either as FMN, hRFK, sRFK or RF substrate). Note that for sRFK samples two different reactions mixtures were prepared; one contained in syringe 1 RFK together with aPNPOx while substrates of both enzymes where placed in syringe 2 (equivalent to sRFK), in the other aPNPOx was alone in syringe 1 and RFK was placed in syringe 2 together its substrates and PNP (sRFK_2_). Final concentrations in the stopped-flow mixture chamber were [PNPOx homodimer] = 0.05 µM, [PNP] = 25 μM, [FLV] = 0.25 µM. Measurements were performed in 50 mM Tris/HCl pH 7.6, 0.3 mM MgCl_2_, 1 mM DTT at 25°C. Data for control holo PNPOx are shown in black, while those for FMN sources (FLV) being free FMN, hRFK, sRFK and sRFK_2_ are respectively shown in red, green, blue and dark purple.

To evaluate the capability of the FMN tightly bound to hRFK to activate aPNPOx, we used a steady-state transfer assay that monitors formation/activation of the holo-form of PNPOx by measuring its ability to transform its PNP substrate into the PLP product (Figure 4A) (Musayev et al., 2003). In these assays, free FMN, hRFK and a fresh mix of sRFK were evaluated as sources of FMN in the activation of aPNPOx. As shown in Figure 4B, the preformed hRFK complex was able to recover 55% of the aPNPOx activity when compared to an equal amount of native PNPOx, while free FMN or FMN produced *in situ* by the RFK activity recovered up to 66% and 72% of the oxidase activity respectively. To identify any potential differential effect of the incubation time on the recovery from aPNPOx to PNPOx, kinetic data were also obtained as saturating PNP substrate concentrations using different incubation times and amounts of FMN to be deliver (Figures 4C, D). In general, pre-incubation hardly modified the recover percentages, but again pointed to a slight improvement of the FMN incorporation into aPNPOx when transferred upon its immediate biosynthesis by RFK over that from bulk solution. It is also worth to note that such recovery percentages are considerably higher than those reported for other systems; in example transference of PLP from PNPOx to acceptor proteins in general ranges between ∼25–40% (Ghatge et al., 2012; Ghatge et al., 2016).

Initial absorbance changes for kinetic traces upon PLP production by aPNPOx after activation by FMN from different sources were also evaluated by stopped-flow spectrophotometry. As shown in Figure 4E, native PNPOx readily produced the PLP-adduct upon mixing, while a lag phase was observed for the remaining samples. This lag phase was considerably shorter for FMN when coming from bulk as compared to from the different RFK sources. It must be potentially related with FMN incorporation into aPNPOx to organize a functional protein plus its release from RFK when bound to it. Noticeably, the lag phase length also differed as a function of the RFK source, with the sRFK sample, that *in situ* generates FMN, showing the shorter one (Figure 4E). Moreover, once the lag phase reached its end, this sample was the only one able to achieve initial rate constants for PLP production in the range of those exhibited by aPNPOx samples reconstituted by bulk FMN.

Finally, since FMN-release is pointed as the rate-limiting step in RFK catalysis (Anoz-Carbonell et al., 2020a), the potential modulation of the RFK activity by the presence of aPNPOx was also evaluated. As shown in Table 2, increasing amounts of aPNPOx produced slight decreases in the *K*_m_^RF^ of RFK while hardly impacting *k*_cat_ values, thus slightly favouring the RFK catalytic efficiency (Table 2). This result suggests that the presence of aPNPOx has an overall positive effect in speeding up the RFK activity limiting-step for FMN-release. Nonetheless, upon further increasing aPNPOx concentrations a slight decrease on *k*_cat_ values was observed, with the consequent deleterious effect on overall catalytic efficiency and pointing to a saturation profile indicative of RFK becoming saturated by aPNPOx.

**TABLE 2:** Impact of the presence of aPNPOx on the RFK steady-state kinetic parameters.

[aPNPOx homodimer] (nM)	*k*_cat_ (min^−1^)	*K*_m_^RF^ (µM)	*k*_cat_/*K*_m_^RF^ (min^−1^.µM^−1^)
0	60 ± 1	2.2 ± 0.3	27 ± 4
12.5	59 ± 2	1.8 ± 0.3	33 ± 5
25	59 ± 1	1.7 ± 0.3	35 ± 6
40	59 ± 3	1.8 ± 0.3	33 ± 6
50	46 ± 2	1.6 ± 0.3	29 ± 5

### 3.3 The RFK and PNPOx conformational landscapes are respectively modulated by their ligation and oligomerization states

Since these characterizations indicated physical interaction between RFK and PNPOx, we moved to evaluate conformations for these proteins that might be relevant for their physiological interaction, namely hRFK and aPNPOx.

**FIGURE 5:**
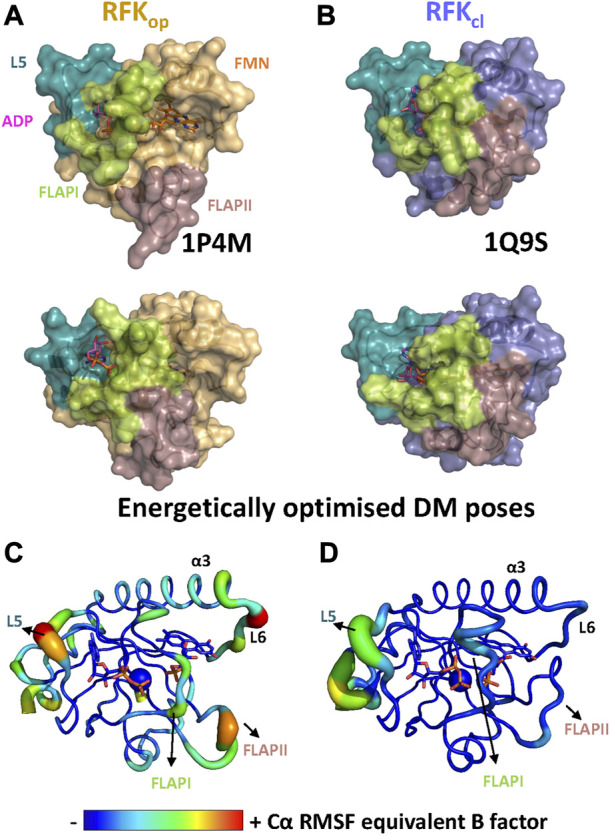
The conformational space of RFK. Comparison of **(A)** the hRFK_op_ crystallographic model (1P4M, top) and one of the representative structures after its MD simulation (bottom), and **(B)** the hRFK_cl_ crystallographic model (1Q9S, top) and one of the representative structures after its MD (bottom). Protein models are shown as light orange and blue-slate surfaces respectively when starting from hRFK_op_ and hRFK_cl_ models. In all cases, key FlapI, FlapII and L5 loops are coloured in lime, dirty violet and green deep teal respectively. ADP and FMN are shown as sticks with carbons in magenta and orange respectively, while Mg^2+^ is shown as a green sphere. The flavin binding site is solvent-accessible in the starting open conformation, but it evolves to more close-like conformations along simulation. Conformational flexibility, as representative Cα RMSF equivalent B factors plotted on the final MD simulation structure of one replicate when using as starting model **(C)** hRFK_op_ and **(D)** hRFK_cl_ states. Only data for one replica are shown. Larger radius of ribbons and particularly warmer colours indicate higher fluctuations. ADP and FMN are shown as sticks and Mg^2+^ as a sphere, all of them are CPK coloured with carbons represented by equivalent B factor colour.

Crystal structures of RFK ternary complexes with the products of the reaction have revealed that open and closed flavin site conformations, hRFK_op_ and hRFK_cl_, can be populated (Figures 5A, B) (Karthikeyan et al., 2003). Therefore, different conformations of the hRFK ligation state might be potential FMN donors to client proteins. MD simulations starting from both crystallographic hRFK_cl_ and hRFK_op_ states were carried out until convergence (Supplementary Figure S1) to produce potential conformations that might act as FMN donors (Figure 5). Most relevant conformational changes when MD started from hRFK_op_ occurred in loops FlapI, FlapII and L5. FlapI and FlapII were displaced towards the isoalloxazine binding cavity, producing intermediate flavin open-closed-like conformations upon reaching equilibrium (Figures 5A, C). In addition, FlapI moved away from L5, contributing to the dynamic break of the Lys20-Asp88 salt bridge and the opening of the ADP/ATP binding site (Figures 5A, C; Supplementary Figure S2). These later changes in conformation were previously described for the equilibration of RFK-free by MD, and appear independent of FlapII closing (Anoz-Carbonell et al., 2020a). On the contrary, hRFK_cl_ hardly showed any significant conformational change, with only minor displacements in L5 and a portion of FlapII (Figure 5B). In agreement, RMSF equivalent B-factors showed hRFK_op_ as more conformational flexible than hRFK_cl_ (Figures 5C, D). Despite its potential displacement, due to its size FlapII showed in general a similar solvent accessible surface area (SASA) in hRFK_cl_ and hRFK_op_ conformations, while ligand RMSD values indicated that FMN remained more stably bound to the protein than ADP during simulation (Supplementary Figure S2B). This latter observation agrees with the adenine of ADP being even able to leave its binding cavity in some hRFK_cl_ and hRFK_op_ replicates, while this was never observed for FMN. Such observations are in line with the proposed catalytic ordered bi-bi mechanism in which ADP would be released before FMN, and further support the hypothesis that FMN is not able to leave the cavity by its own (Anoz-Carbonell et al., 2020a). It is also worth to note that the 25-PTAN-28 active site motif and the Glu78 catalytic residue hardly changed in conformation along MD simulations, being also their conformation quite similar and robust in the different protein ligation states (Figure 5; Supplementary Figure S2B). Therefore, these simulations agreed with the RFK ligation state highly influencing its conformation, and showed that while open conformations of adenine and flavin sites are highly populated for RFK-free, several closed-like conformations might be favoured when in ternary complex with the products of the reaction.

Regarding *H. sapiens* PNPOx, the conformational flexibility was evaluated by subjecting its crystallographic derived aPNPOx monomer and homodimer models to MD simulations. Noticeably, RMSF and RMSF equivalent B factors for the protein monomer in the monomeric state showed regions of particular high mobility at the 185–195 α6 helix and the 195–198 β-turn following it at the interdomain cap that folds out of the PNPOx and PNP_phzG_C domains and closes one of the edges of the FMN binding site (Supplementary Figure S3, left). Other regions showing flexibility were the 81–87 and 239–242 β-turns respectively sited at the PNPOx and PNP_phzG_C domains. However, the dynamics of the interdomain cap turned out to be considerably restricted when evaluating the homodimer state (Supplementary Figure S3, right), probably as a consequence of the produced inter-subunit interactions. In both structures, but particularly in the homodimer envisaged to act as FMN acceptor, residues and regions involved in FMN and PLP binding remained relatively immobile.

### 3.4 Models for the PNPOx:RFK coupling envisage potential channelling organizations

Molecular docking/dynamics simulations were subsequently conducted to produce potential binding modes between relevant states of RFK and PNPOx for FMN transfer. The direct docking of RFK_op/cl_ crystallographic structures onto the PNPOx homodimer identified interacting surfaces hot-spots that in general sit around the FMN binding site in PNPOx, while RFK_op_ and RFK_cl_ showed particular hot-spots regions (Supplementary Figure S4). FlapI and FlapII were major contributors for RFK_op_ coupling to PNPOx, but their contribution became marginal for couplings involving RFK_cl_ where β1, α2, L2, α3, and/or L6 regions replaced them. Hot-spots on PNPOx concentrated around its FMN binding site at the cleft between both protomers, but contributions of the different structural elements also depended on the conformation of the donor protein. RFK_op_:PNPOx poses maximizing hot-spots hardly produced orientations suitable for transference between donor and acceptor FMN binding sites, but poses with the best rankings in energy did provide favourable conformations for the transfer (Supplementary Figure S4). Noticeably, initial RFK_cl_:PNPOx couplings were not enriched in poses with adequate orientations for FMN transfer, since FlapI and FlapII shielding the FMN cofactor were away from the PNPOx site for the cofactor. Therefore, additional docking poses for these interactions were generated using the optimized RFK_cl_ models that were then evaluated considering selected hot-spots to produce RFK_cl_:PNPOx poses with more suitable orientations for FMN transference.

**FIGURE 6:**
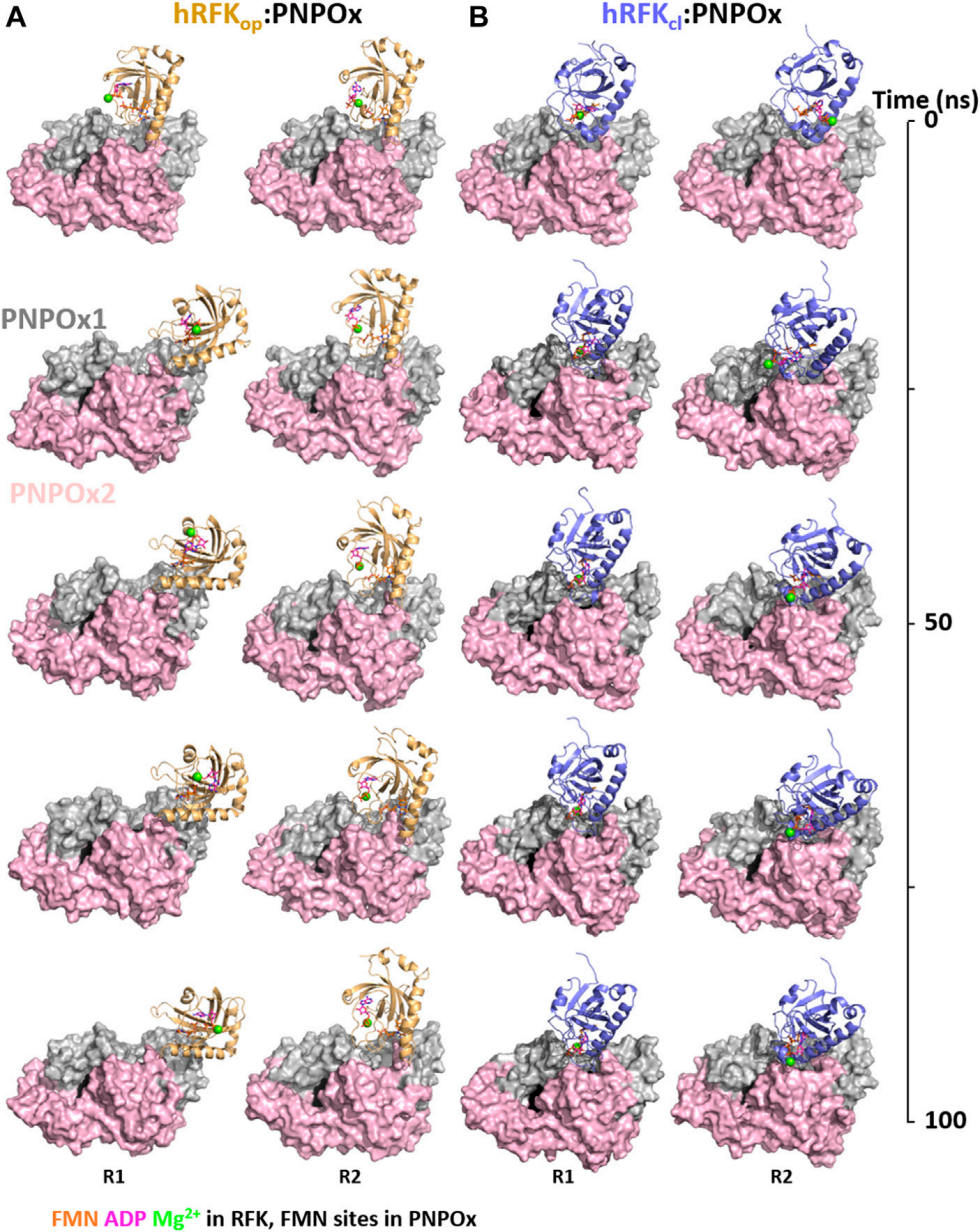
Energetic optimization of RFK:PNPOx docking models by MD simulations. Representation of the progress over time of the MD simulation of selected **(A)** hRFK_op_:PNPOx and **(B)** hRFK_cl_:PNPOx docking complexes. For each complex two representative replicas are shown (R1 and R2 in Supplementary Figures S5–S7), corresponding to two different starting complexes obtained by replacing RFK from the selected docking pose [less energy pose or best favouring restrictions respectively for **(A)** and **(B)**] with two different conformations of hRFK_op_ (in A) or hRFK_cl_ (in B) along their respective MD simulations in ternary complexes with the products of the reaction (Figure 5; Supplementary Figures S1, S2). RFK structures starting from hRFK_op_ and hRFK_cl_ structures are coloured in light orange and blue-slate respectively, one of the PNPOx protomers is coloured in grey and the other in light pink. RFK is shown in cartoon, while PNPOx is shown in surface. ADP and FMN are shown as sticks with carbons in magenta and orange respectively, while Mg^2+^ is shown as a green sphere. In order to locate the active site in PNPOx, the predicted position for the FMN molecule (coming from aligned PDB 1NRG) is shown in black spheres.

**FIGURE 7:**
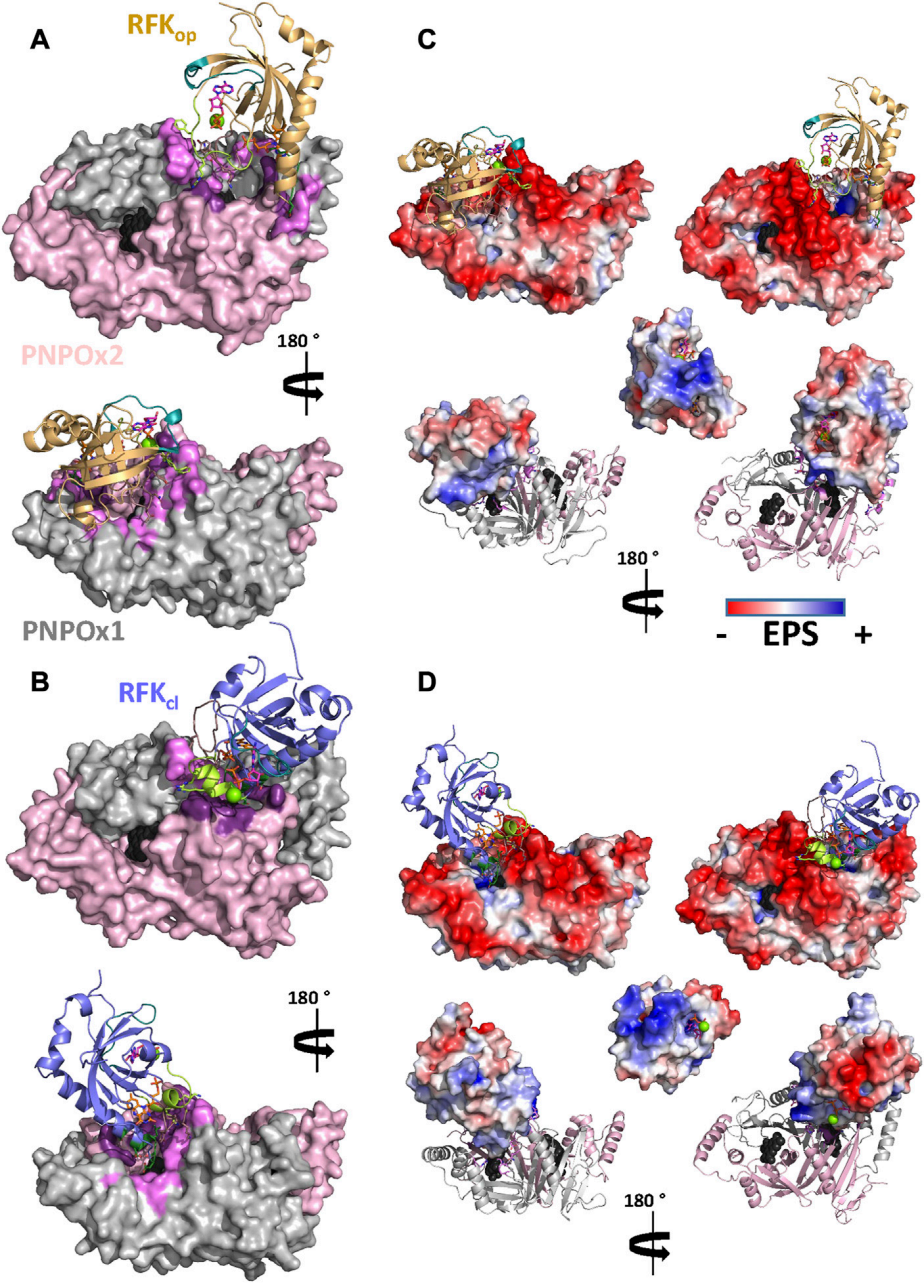
Molecular coupling of RFK to aPNPOx. Representative final interaction models for the coupling of **(A)** hRFK_op_ and **(B)** hRFK_cl_ to aPNPOx. Contribution of ESP to the **(C)** hRFK_op_:aPNPOx and **(D)** hRFK_cl_:aPNPOx interaction models. Top, bottom and central panels respectively show the ESP for the aPNPOx dimer while hRFK is in cartoon, for RFK while aPNPOx is in cartoon and for the RFK at the surface docking into aPNPOx. Unless ESP is shown, hRFK_op_ and hRFK_cl_ molecules are respectively shown as light orange and blue-slate cartoons, with FlapI, FlapII and L5 loops coloured in lime, dirty violet and green deep teal respectively, and contact points with PNPOx at L6 and/or α3 in green forest. ADP and FMN are shown as sticks with carbons in magenta and orange respectively, while Mg^2+^ is shown as a green sphere. The two PNPOx protomers are shown in surface in panels **(A)** and **(B)** and in cartoon in panels **(C)** and **(D)** and respectively coloured in grey and light pink, with hot spots for the RFK interaction highlighted in pale and dark purple. In all cases the two active sites in the PNPOx dimer are located by the predicted position for the FMN molecule (coming from aligned PDB 1NRG) in black spheres. In both cases data are shown for representative replicate R2 (Supplementary Figures S6, S7).

**FIGURE 8:**
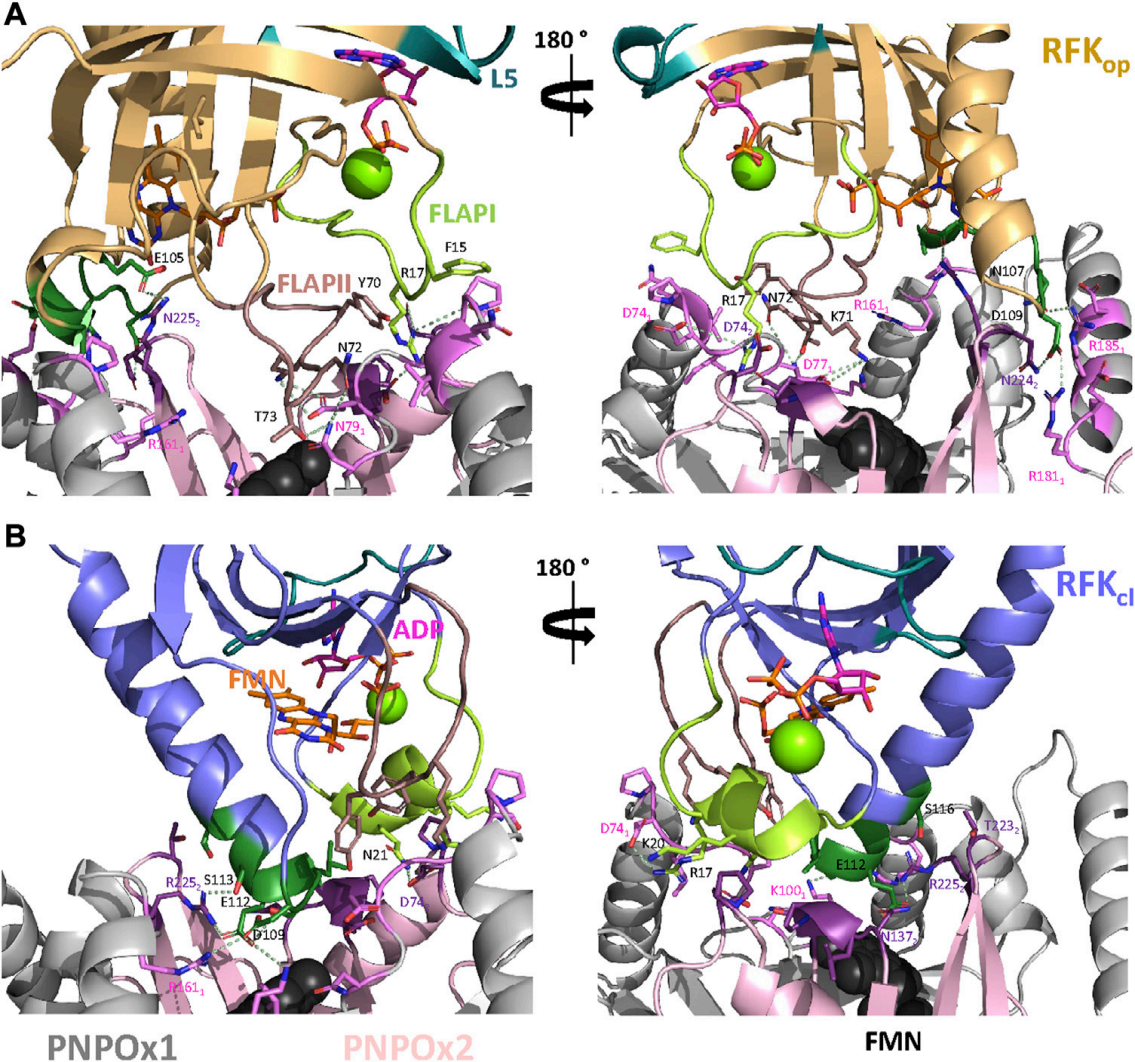
Key residues at the putative RFK:PNPOx interface. Representative key interactions at the protein-protein interface for models of the binding of **(A)** hRFK_op_ and **(B)** hRFK_cl_ to aPNPOx. Key residues for the interaction are labelled in black for hRFK and according to protomer colour code for PNPOx. Colour codes as in Figure 7. Representative electrostatic couplings are highlighted by dashes in pale green. In both cases data are shown for representative replicate R2 (Supplementary Figures S6, S7).

These docking models provided rigid-body couplings with different relative conformations and orientations between the FMN binding sites at donor and acceptor proteins, which might be populated during their dynamic interaction. Nonetheless, these models neither considered conformational flexibility of individual proteins as a factor for mutual adaptation, nor the fact that hRFK_op_ (slightly open in close-like conformations) and hRFK_cl_ (with entrapped FMN) structures showed significant conformational differences even after equilibration (Figure 5). To contemplate that the interaction should surely encompass several hRFK conformations, two poses from each rigid-body docking complex (favouring the energy and/or hot-spots ranking) were selected and their RFK molecule was replaced by hRFK_op/cl_ models coming from at least two different optimized MD frames (Figure 5). In this way four different hRFK_op_:aPNPOx and hRFK_cl_:aPNPOx structures were produced, which were herein evaluated as replicates and further optimized by MD simulations up to convergence (Figure 6; Supplementary Figures S5–S7). In general, trajectories for the last 50 ns within each replicate clustered in a single relative conformation of hRFK regarding the aPNPOx homodimer (Figure 6; Supplementary Figures S6, S7). Nonetheless, some hRFK_op_:aPNPOx replicates showed the ability of hRFK_op_ to rotate on the PNPOx surface and to get displaced to preferentially bind to one of the aPNPOx protomers, modulating the residues contributing to the interaction and the distance between the donor and acceptor sites of FMN within the interacting complex (Figure 6; Figure 7A; Supplementary Figures S6, S8). On the contrary, changes in relative orientations between interacting partners in the hRFK_cl_:aPNPOx replicates were minor along the MD simulations, and, if any, they reduced proximity between FMN donor and acceptor binding sites and further sit hRFK_cl_ right at the cleft between the aPNPOx protomers (Figure 6; Figure 7B; Supplementary Figures S7, S8). Both hRFK_op_:aPNPOx and hRFK_cl_:aPNPOx models show electrostatics couplings of positive ESP regions on RFK and negative ones on the PNPOx homodimer, as well as good surface complementarities and formation of salt bridges and H-bonds across protein-protein interfaces (Figures 7, 8; Supplementary Figures S9, S10). Despite the fact that FMN binding sites between donor and acceptor proteins situated at a larger distance in hRFK_op_:aPNPOx models than in hRFK_cl_:aPNPOx complexes (Supplementary Figures S9B, S10B), those holding hRFK_op_ show a continuous cavity that connects the cofactor binding sites of donor and acceptor. On the contrary, such connexion is blocked in hRFK_cl_:aPNPOx structures, since FlapI and FlapII entrap FMN within RFK. These observations are in line with the very different interaction modes with PNPOx envisaged from our ITC results (Figure 1).

## 4 Discussion

Around 25% of flavoproteins in *H. sapiens* use FMN as cofactor, or bind it either as substrate/product or modulator (Supplementary Table S1) (Gudipati et al., 2014; Pacheco-Garcia et al., 2021). Moreover, FMN also happens to be the precursor of the FAD cofactor required for the remaining flavoproteome. Flavoproteins are typically biosynthesized as apo-forms, being later converted to their active holo-forms through the acquisition of FMN or FAD. In particular, FMN is produced by RFK, and the traditional paradigm stipulates that for its transfer it must be released from RFK into the bulk and then taken by the client apo-proteins. This rises an intriguing question of how the low concentration envisaged for cellular free flavins supports cofactors for the activation of the around 100 human competing apo-flavoproteins. Such observations, together with RFK being inhibited by its FMN product and keeping it strongly bound, envisaged the possibility of cells solving this problem by shielding the FMN through the formation of a complex between RFK and client apo-proteins for productive direct cofactor transfer (Anoz-Carbonell et al., 2020a). Our ITC results show that RFK is able to interact with affinities in the low micromolecular range with one of its potential FMN client apo-proteins, PNPOx (Figure 1). Such interaction is further confirmed by increasing thermal stability in both apo-proteins upon incubation with the potential partner, as well as by changes in RFK efficiency when aPNPOx is present in the solution (Figure 2; Tables 1, 2). *In vitro* and in the absence of RFK, aPNPOx is indeed able to incorporate FMN directly from the bulk; nonetheless, changes in the kinetics for FMN incorporation when hRFK acts as the FMN source are evident (Figure 3). Thus, FMN incorporation into aPNPOx becomes regulated in a saturation profile manner that slightly enhances FMN incorporation rates at flavin concentrations close to those equimolecular with the acceptor sites (Figure 3). This behaviour agrees with channelled processes usually being only slightly enhanced, or even slow down, regarding non-channelled ones (Poshyvailo et al., 2017), because their main objective is protecting intermediates from degradation or competing pathways. This in turn results in more efficient reactions, while also preventing cells from the effect of unstable or cytotoxic intermediates as well as from undesired interactions of ligands (Poshyvailo et al., 2017; Zhang and Fernie, 2021; Zhang and Fernie, 2022). In our particular case, the strong binding of the FMN product within hRFK and its effect as a competitive inhibitor of the RF substrate would provide a safe and efficient pathway for productive activation of apo-FMN-dependent proteins, envisaging that the interaction of hRFK with its client apo-proteins might favour FMN release (Anoz-Carbonell et al., 2020a). As far as we know, no equivalent data have been reported yet for the study of FMN transfer between proteins. Nonetheless, several groups have already addressed the study of potential PNPOx transient interactions to several of its PLP product client proteins, determining affinity parameters that lay in range of that here determined for the here evaluated RFK:PNPOx interactions (Cheung et al., 2003; Ghatge et al., 2016; Al Mughram et al., 2022). Thus, the binding parameters, together with the capability of hRFK and, particularly, sRFK to activate aPNPOx, agree with formation of stable transient interactions between both proteins (Figures 1, 4). However, they also suggest an intricate thermodynamic binding and transference landscape that is very sensitive to particular RFK and PNPOx ligation states, as well as to the environment. Complexity in these interactions is expected, particularly when considering the existence of equilibria involving the partial release of bound ligands from hRFK/sRFK to the bulk solution or the transfer of FMN to aPNPOx, together with the facts that the functional unit of PNPOx is a homodimer and the RFK catalytic cycle relies on sequential large conformational changes in the loops giving access to its substrates/products binding cavities (particularly FlapI, FlapII and L5) (Anoz-Carbonell et al., 2020a). This generates a complex network of RFK conformational states that are highly dependent on differential features from binding thermodynamics, kinetics and cooperation between substrates and/or products, which in addition is reported as highly species-specific when evaluating enzymes holding RFK activity in different species (Sebastián et al., 2017; Sebastián et al., 2018b; Anoz-Carbonell et al., 2020a). Therefore, different significant complex arrangements might be produced for different RFK ligation and conformational states (open/closed-like/closed transitions) (Figure 5), producing RFK:PNPOx assemblies characterized by different interaction thermodynamic parameters. In agreement we have also observed divergent behaviours between hRFK and sRFK in aPNPOx activity recovering (Figure 4), which indicate that RFK conformation modulates its capability for FMN transfer assemblies to the acceptor.

The availability of structures of RFK and PNPOx enabled us to undertake docking and MD simulations to predict likely near-native structures of the complex, as well as the influence of the RFK conformation on such assemblies. The here produced molecular interaction models for potential hRFK:aPNPOx associations envisaged that, particularly, the conformations of FlapI and FlapII loops in hRFK considerably impact the coupling between both proteins (Figures 6–8). They also show loops at the PNPOx FMN binding site cavity entrance contributing to protein-protein fitting. These observations support the possibility of transient “loop-mediated” interactions between both proteins contributing to the stabilization of assemblies with different probabilities to release and transfer FMN from its tight binding site in RFK to aPNPOx. Furthermore, this hypothesis is supported by RFK potentially exhibiting binding sites of different affinity upon interaction with PNPOx (Figure 1). Altogether, the models here produced support the RFK-PNPOx interaction from the structural point of view, while indicating that interaction modes will highly depend on hRFK conformation. In this context, hRFK_cl_ conformations, despite favouring proximity between FMN binding cavities of donor and acceptor proteins, would require subsequent conformational changes for flavin delivery (Supplementary Figures S10). On the contrary, open-to-closed-like conformations would show both cavities being connected with binding sites being at larger distances (Supplementary Figures S9). Such observations would support *in vivo* aPNPOx activation being partly due to the establishment of the equilibrium of FMN binding to the donor and acceptor proteins as a consequence of their conformations, and would imply that not all FMN might be expected to be transferred from the donor protein to the acceptor protein. Moreover, channelling mechanisms involving protein-protein interactions (PPI) will surely be physiologically controlled under well-defined conditions, such as a given form of the cofactor, particular conformations of the donor and acceptor proteins, or even will likely require some auxiliary factors or proteins. In fact, among the few examples reported for cofactor transfer, most involve accessory proteins. Such behaviour has been reported for the insertion of the molybdenum cofactor into apo-trimethylamine-oxide reductase (TorA), where the auxiliary chaperone TorD enhances the direct molybdenum cofactor transference from the protein synthesizing it, MobA, to the client apo-TorA by favouring physical interplay between both proteins (Genest et al., 2008; Tiedemann et al., 2022). Another of such examples is the NorQ/D-facilitating system for the insertion of the non-heme FeB cofactor into bacterial nitric oxide reductase (Kahle et al., 2018). In this context, it is worth noting that RFK, as well as its FMN product, are widely involved in protein-protein and protein-DNA/RNA networks (Supplementary Figure S11A; Supplementary Table S1). Of note is the particular interaction of RFK with the Tumor Necrosis Factor Receptor 1 (TNFR1), an adapter protein that enhances the assembly of FAD to NADPH oxidases activating the production of reactive oxygen species (Yazdanpanah et al., 2009). As the RFK:TNFR1 interaction, the remaining nodes of the BioGrid RFK interaction network do not directly envisage flavin transfer to client apo-proteins, but they contribute to different cellular processes whose regulation is critical to prevent pathological situations (Hirano et al., 2011; Barile et al., 2013; Chen et al., 2020). The BioGrid PNPOx network envisages more partners for this protein. Nonetheless, again in general they are not PLP acceptors, being a bunch of them cell membrane carrier proteins or modulators of other proteins (Supplementary Figure S11B; Supplementary Table S1). However, most of these interactors are still poorly characterized, particularly when related to potential partners. As a consequence, so far we cannot rule out the possibility of auxiliary proteins also contributing to enhance the FMN transfer from RFK to PNPOx, as well as of PLP from PNPOx to the client protein.

In conclusion, we provide experimental data that confirm the physical interaction between RFK and PNPOx, show the ability of aPNPOx to acquire FMN from hRFK/sRFK, and predict likely interaction modes that might contribute to transference of the tightly bound FMN from hRFK to aPNPOx as well as to its regulation. Formation of these complexes might be a way to prevent FMN from coming into contact with other cellular proteins or molecules. Therefore, hereinafter, the relevance of flavin cofactors in several cellular processes, as well as in the onset of pathologies, should also be looked at from the perspective of whether delivery mechanisms involving physical PPIs are working appropriately. *In vivo* inborn errors that might disrupt such PPIs could prevent either FMN incorporation or affect the adequate regulation of flavin homeostasis with severe consequences. Proving the formation of RFK:PNPOx complexes is a step forward for the understanding of mechanisms where flavin transference must occur, as well as of how FMN and FAD synthesis are regulated in the cell to match the actual needs in light of the low concentrations of free flavins and their affinities when bound to flavoproteins. In addition, this study also paves the bases to evaluate some PNPOx mutations associated with pathological disorders within the context of the FMN cofactor incorporation. Moreover, our results place the notion that binding energetics and cooperativity are fundamentally linked with the dynamic nature of RFK and PNPOx conformational ensembles, as well as conected at the molecular level stability of RFK-PNPOx PPIs with intracellular fluctuations of FMN levels.

## Data Availability

The original contributions presented in the study are included in the article/Supplementary Material, further inquiries can be directed to the corresponding author.
