# Supplementary material for: Riboflavin kinase and pyridoxine 5′-phosphate oxidase complex formation envisages transient interactions for FMN cofactor delivery
Source: Front Mol Biosci. 2023 Mar 28;10:1167348. doi: 10.3389/fmolb.2023.1167348 (PMC10086132; doi:10.3389/fmolb.2023.1167348)
Supplement: Supplementary file 1 [file DataSheet1.pdf]

Supplementary Material

Riboflavin kinase and pyridoxine 5'-phosphate oxidase complex formation envisages transient interactions for FMN cofactor delivery

Maribel Rivero[1,2], Sergio Boneta[1,2], Nerea Novo[1,2], Adrián Velázquez-Campoy[1,2,3,4,5], Victor Polo[2,6], Milagros Medina[1,2,5]

[1]Departamento de Bioquímica y Biología Molecular y Celular, Facultad de Ciencias, Universidad de Zaragoza, Zaragoza, Spain.

[2]Instituto de Biocomputación y Física de Sistemas Complejos (BIFI), Universidad de Zaragoza, Zaragoza, Spain.

[3]Instituto de Investigación Sanitaria Aragón (IIS Aragón), Zaragoza, Spain

[4]Centro de Investigación Biomédica en Red en el Área Temática de Enfermedades Hepáticas y Digestivas (CIBERehd), Madrid, Spain

[5]Group of Biochemistry, Biophysics and Computational Biology 'GBsC' (BIFI, Unizar) Joint Unit to CSIC, Zaragoza, Spain

[6]Departamento de Química Física, Universidad de Zaragoza, Zaragoza, Spain.

*** Correspondence:** Milagros Medina. Departamento de Bioquímica y Biología Molecular y Celular. Facultad de Ciencias. Pedro Cerbuna 12. Universidad de Zaragoza. 50009-Zaragoza. Spain. Fax: +34 976 762123; Phone: +34 976 762476
mmedina@unizar.es

Running title: Protein-Protein interactions in FMN delivery

Journal: Frontiers in Molecular Biosciences

1 Supplementary Figures and Tables

1.1 Supplementary Figures

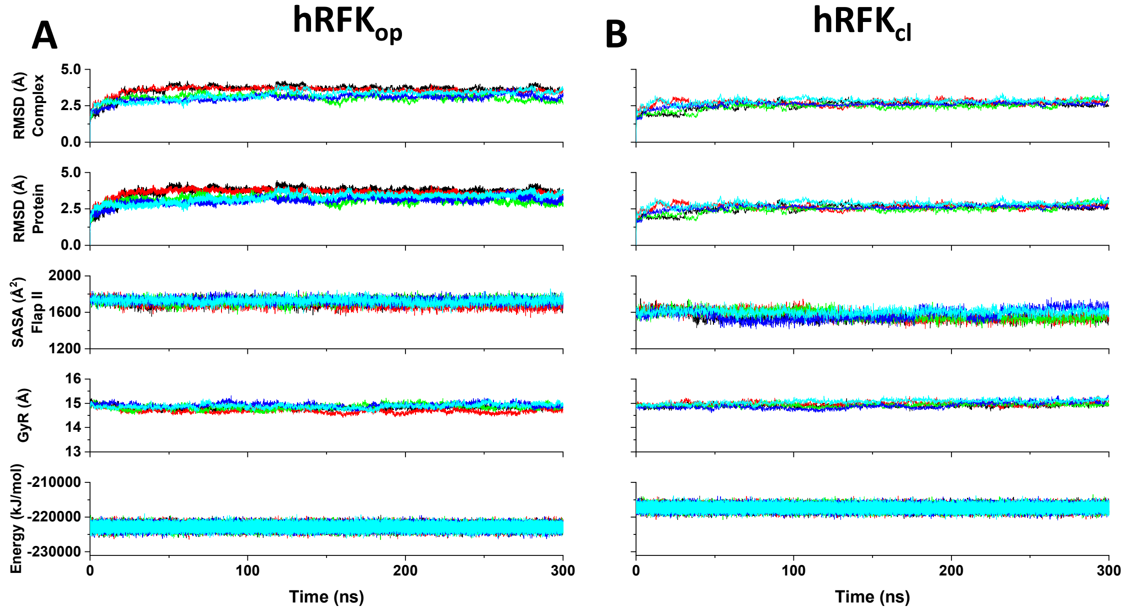

Figure S1. Quality parameters for the MD simulations of RFK in complex with the products of its reaction. Simulations starting in the named **(A)** $hRFK_{op}$ and **(B)** $hRFK_{cl}$ conformational states. Trajectories are shown for root mean square deviation (RMSD) of overall structures and of polypeptidic chain, solvent accessibility surface area (SASA) for FlapII, radius of gyration (GyR) and global energy of the simulated system. Data are shown for the MD simulations of five replicas, with parameters corresponding to the same replicate shown in the same colour in each panel.

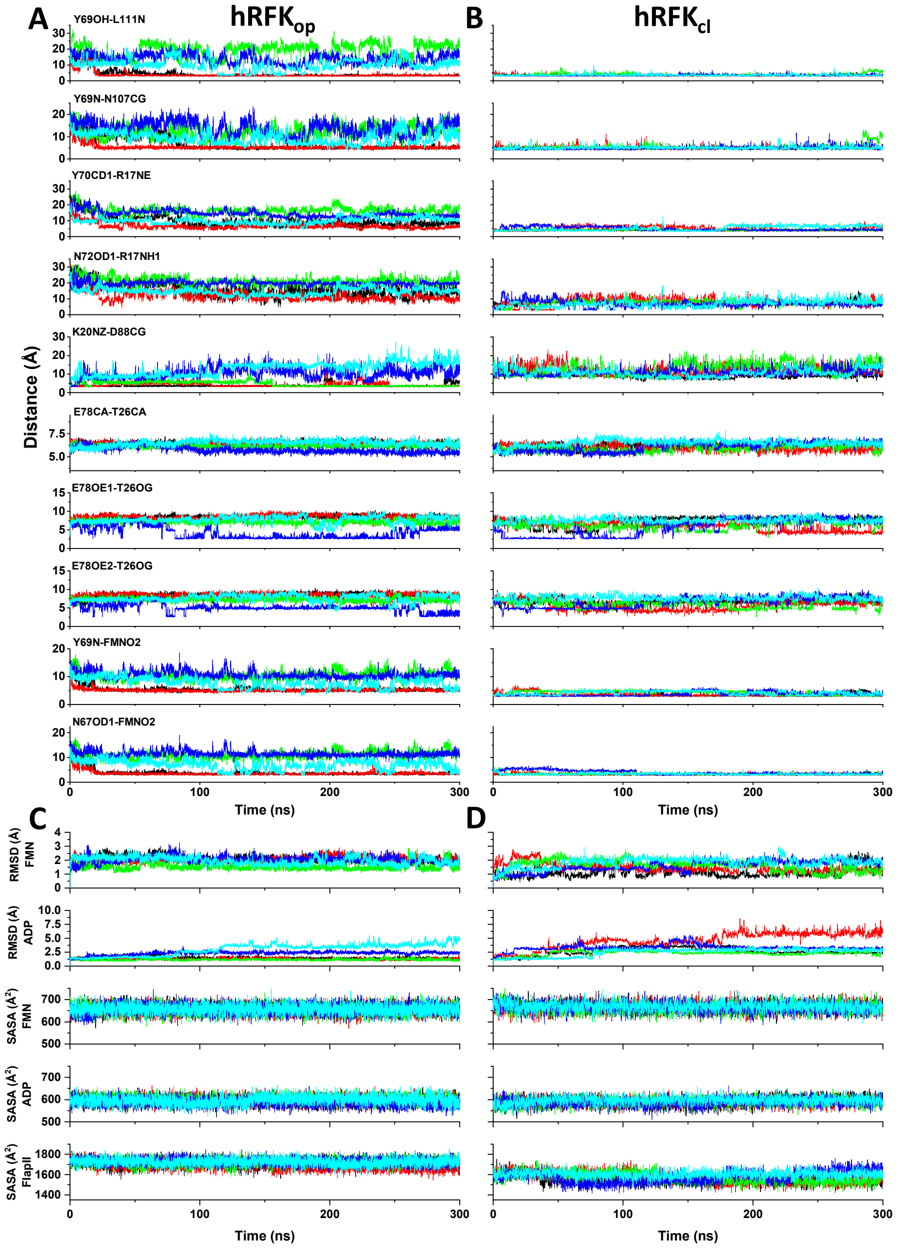

Figure S2. Impact of the starting conformation of RFK in complex with the products of its reaction on the MD trajectories of selected features. Evolution of selected distances in **(A)** hRFK$_{op}$ and **(B)** hRFK$_{cl}$. Evolution of RMSD and SASA for FMN and ADP products and FlapII in **(C)** hRFK$_{op}$ and **(D)** hRFK$_{cl}$. Trajectories are shown for the 300 ns MD simulations of five replicas, with parameters corresponding to the same replicate shown in the same colour.

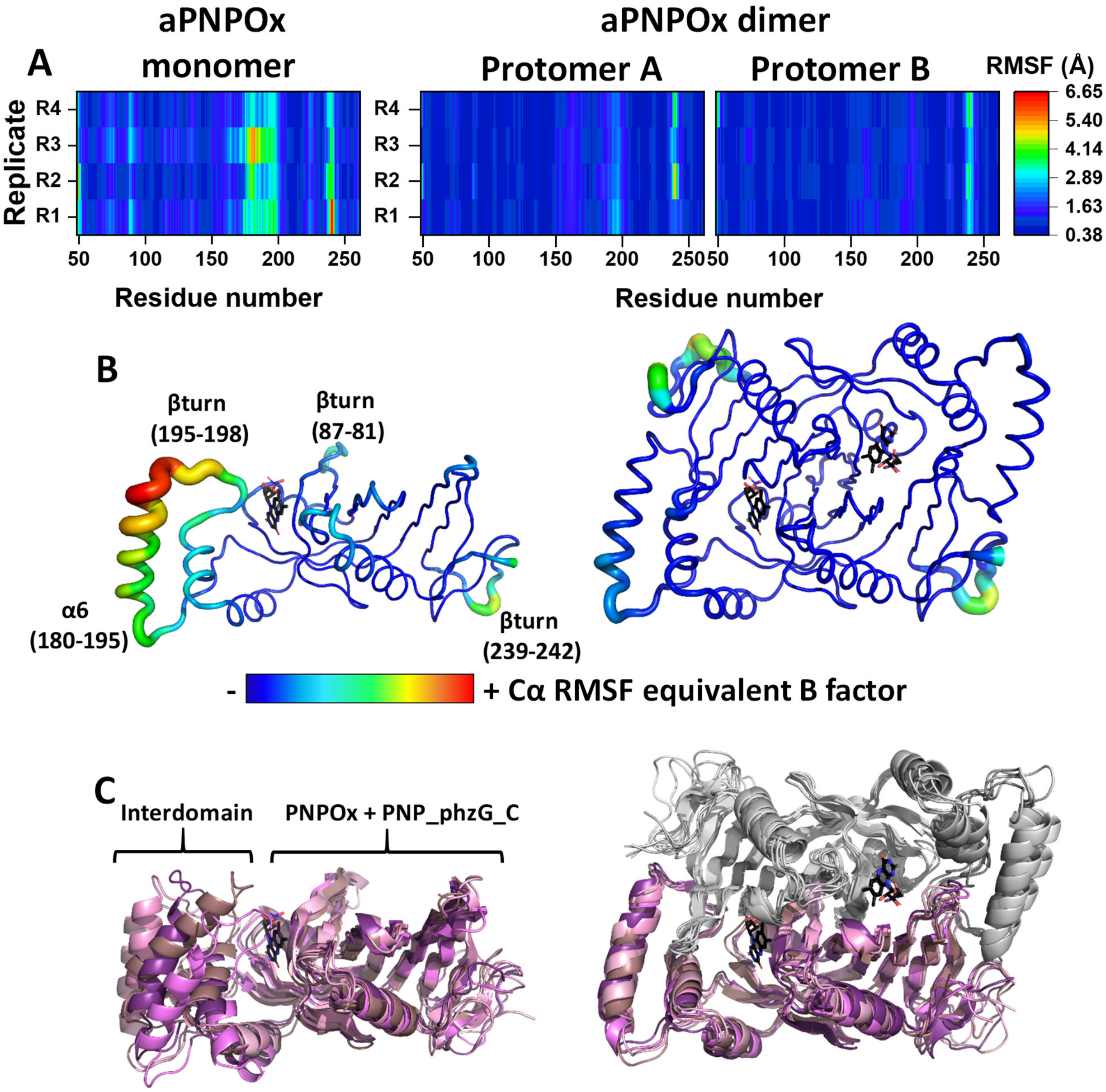

Figure S3. The conformational space of aPNPOx in monomer (left panels) and homodimer (right panels) states. **(A)** Heat map of the Cα RMSF in four replicas of each MD simulations. **(B)** Plot of representative Cα RMSF equivalent B factors on the final MD simulation structure for one of the replicates. Wider radii of ribbons and warmer colours indicate higher fluctuations. **(C)** Cartoon overlapping of final structural models obtained in the five replicates of the MD simulations. The different final structures are shown in pink scales. In the case of the homodimer one of the protomers is shown in grey for clarity. In all cases MD simulated structures are apo-forms, but FMN molecules are shown as CPK coloured sticks with carbons in black to visualize their binding site positions.

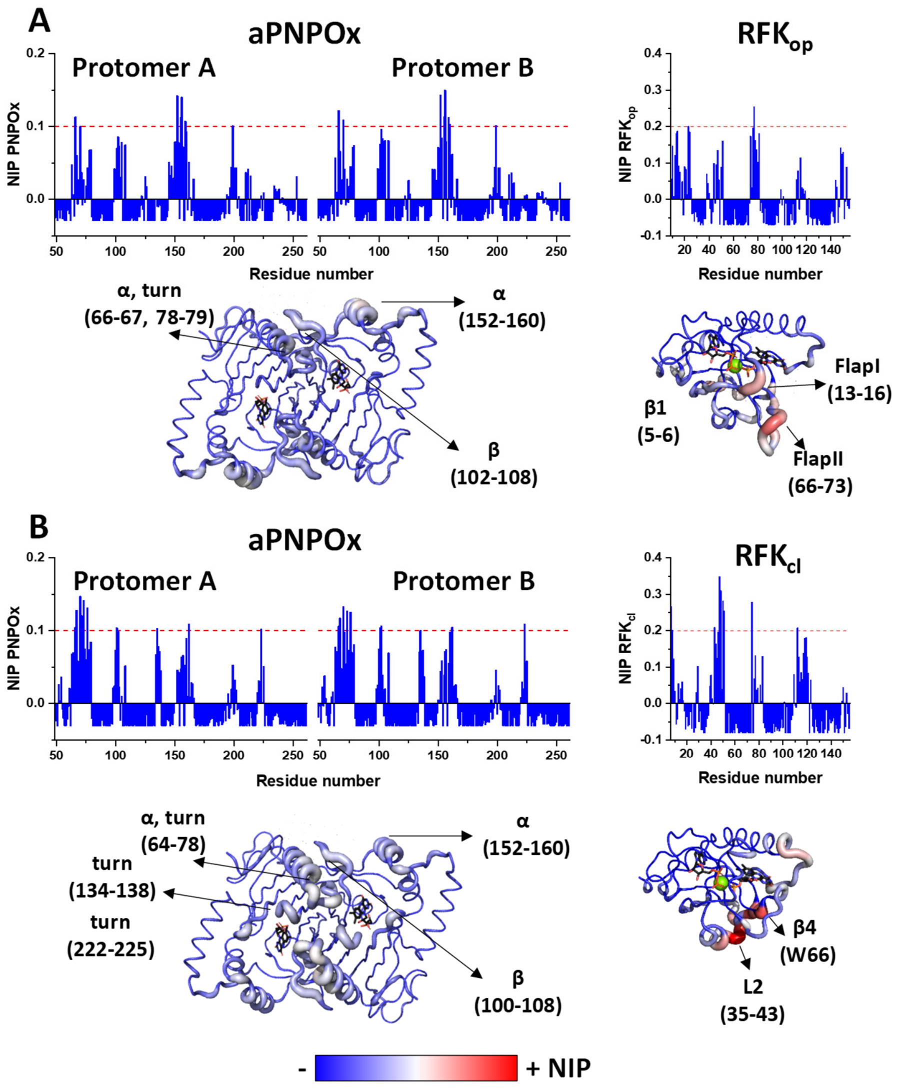

Figure S4. Docking of hRFK$_{op}$ (1P4M) and hRFK$_{cl}$ (1Q9S) crystal structures to PNPOx crystallographic model. Summary for coupling models: (A) RFK$_{op}$:PNPOx and (B) RFK$_{cl}$:PNPOx. For each complex left panels show Normalized interface propensity (NIP) values by residue (top) for PNPOx (left) and RFK (right), as well as NIPs equivalent B factors on their structural models (bottom). Increases in NIPs on models are shown from thinner to thicker and from blue-to-white-to-red (-0.08-0.35 range). For the visualization of ligand ADP and FMN sites, molecules are shown in black lines according to the position they occupy in the crystal structures, while Mg^{2+} position is shown as a green sphere.

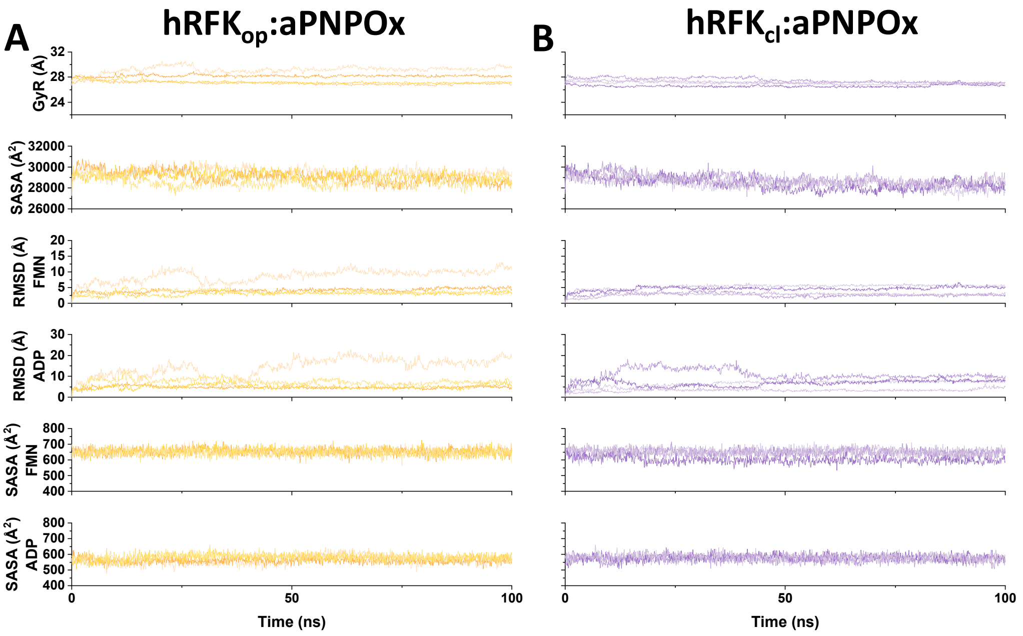

Figure S5. Quality parameters for the MD simulations of hRFK:aPNPOx interaction models. Trajectories are shown in **(A)** hRFK$_{op}$:aPNPOx and **(B)** hRFK$_{cl}$:aPNPOx complexes for GyR, overall SASA, and RMSD and SASA for FMN and ADP within RFK. Trajectories are shown for the 100 ns MD simulations of four representative replicas, with parameters corresponding to the same replicate shown in the same colour.

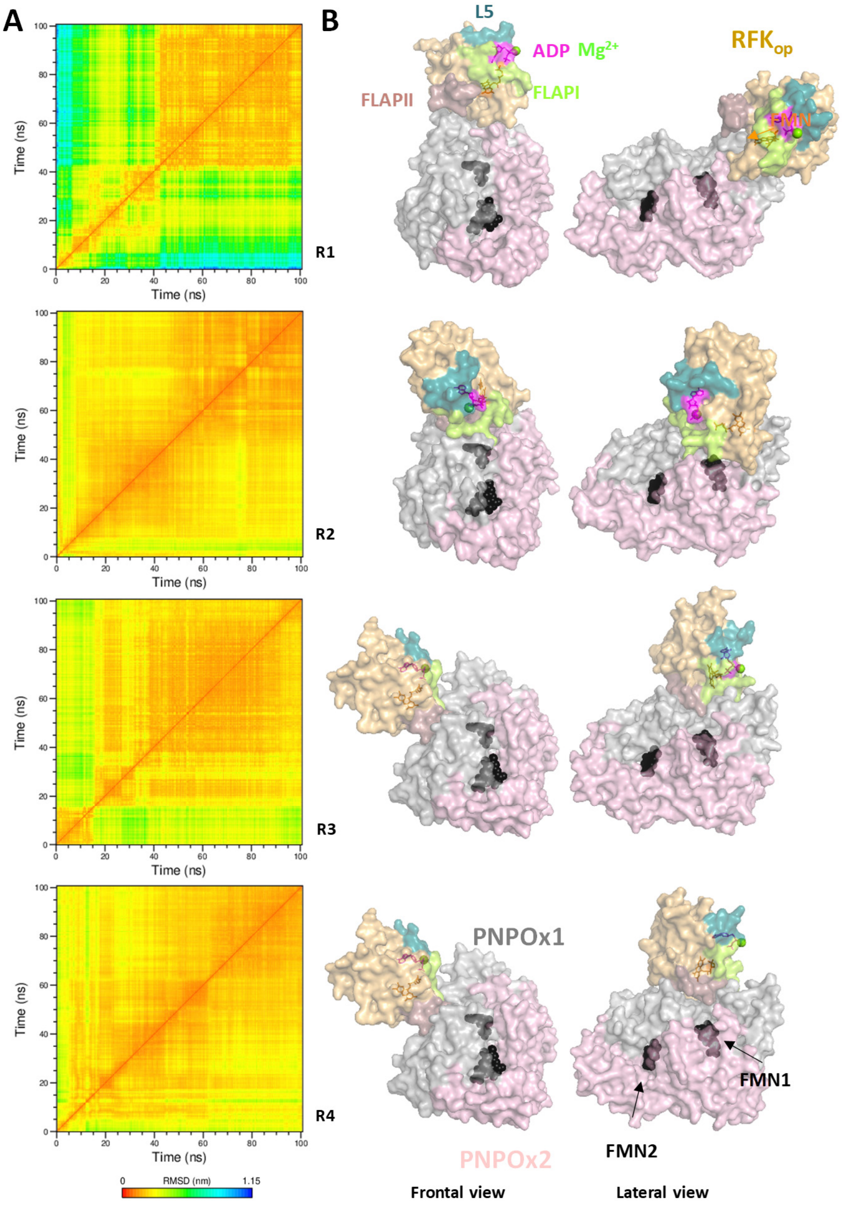

Figure S6. MD simulations of hRFK$_{op}$:aPNPOx putative interaction models. (A) Heat maps representing pairwise RMSD (Å) calculated for the 100 ns simulations. **(B)** Frontal and lateral views for the equilibrated complexes at the end of each replicate MD simulation. The four representative replicates (R1-R4) correspond to different starting complexes obtained by replacing RFK by two different conformations of hRFK$_{op}$ along its MD simulations in ternary complex with the products of its reaction (Figures 5, S1 and S2) in the docking pose of less energy for replicates R1 and R2 and in the docking pose best favouring NIPs (twenty-first in energy ranking) for replicates R3 and R4. hRFK$_{op}$ is shown in light orange surface, with FlapI, FlapII and L5 loops coloured in lime, dirty violet and green deep teal respectively. ADP and FMN are shown as sticks with carbons in magenta and orange respectively, while Mg^{2+} is shown as a green sphere. The two PNPOx protomers are respectively coloured in grey and light pink. In all cases the two active sites in the PNPOx homodimer are located by the predicted position for the FMN molecule (coming from aligned PDB 1NRG) in black spheres.

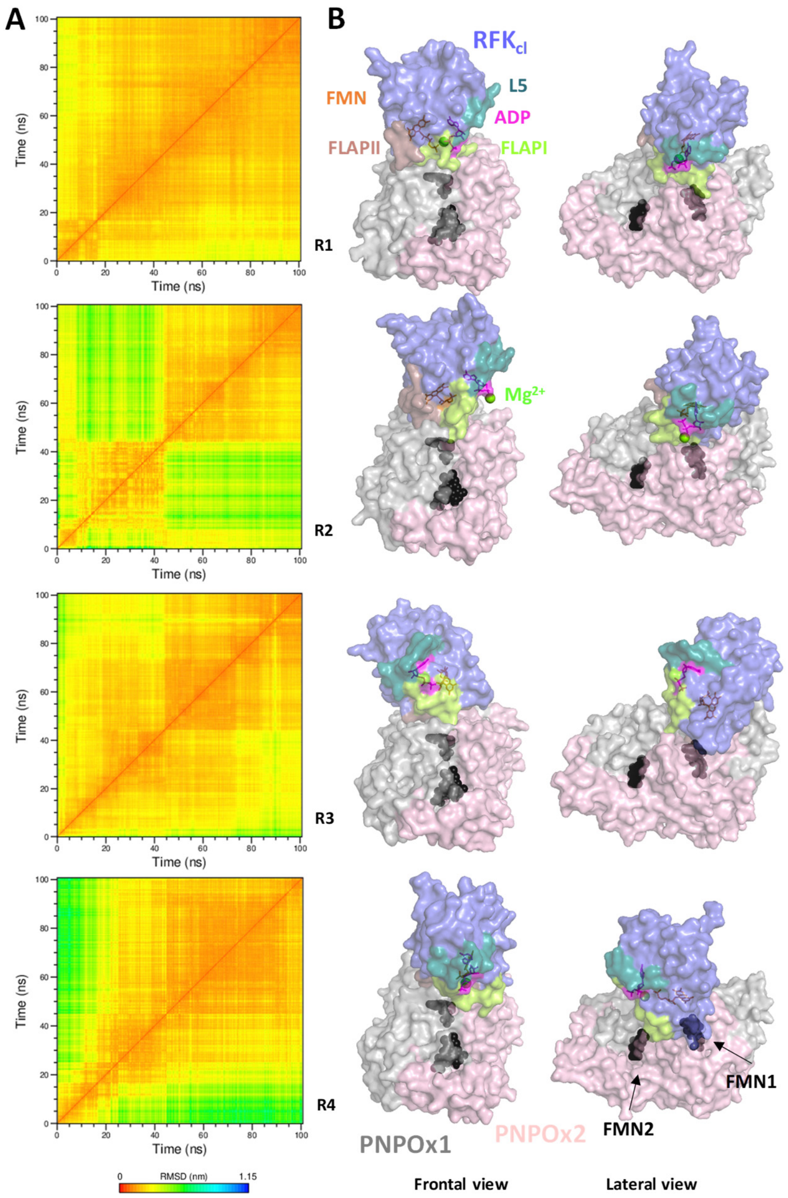

Figure S7. MD simulations of hRFK$_{cl}$:aPNPOx putative interaction models. (A) Heat maps representing pairwise RMSD (Å) calculated for the 100 ns simulations. **(B)** Frontal and lateral views for the equilibrated complexes at the end of the MD simulation. The four representative replicates (R1-R4) correspond to different starting complexes obtained by replacing RFK by two different conformations of hRFK$_{cl}$ along its MD simulations in ternary complex with the products of its reaction (Figures 5, S1 and S2) in the docking poses ranking energy+restrictions in position 1 for replicates R1 and R2 and 7 for R3 and R4. hRFK$_{cl}$ is shown in blue-slate surface, with FlapI, FlapII and L5 loops coloured in lime, dirty violet and green deep teal respectively. ADP and FMN are shown as sticks with carbons in magenta and orange respectively, while Mg^{2+} is shown as a green sphere. The two PNPOx protomers are respectively coloured in grey and light pink. In all cases the two active sites in the PNPOx homodimer are located by the predicted position for the FMN molecule (coming from aligned PDB 1NRG) in black spheres.

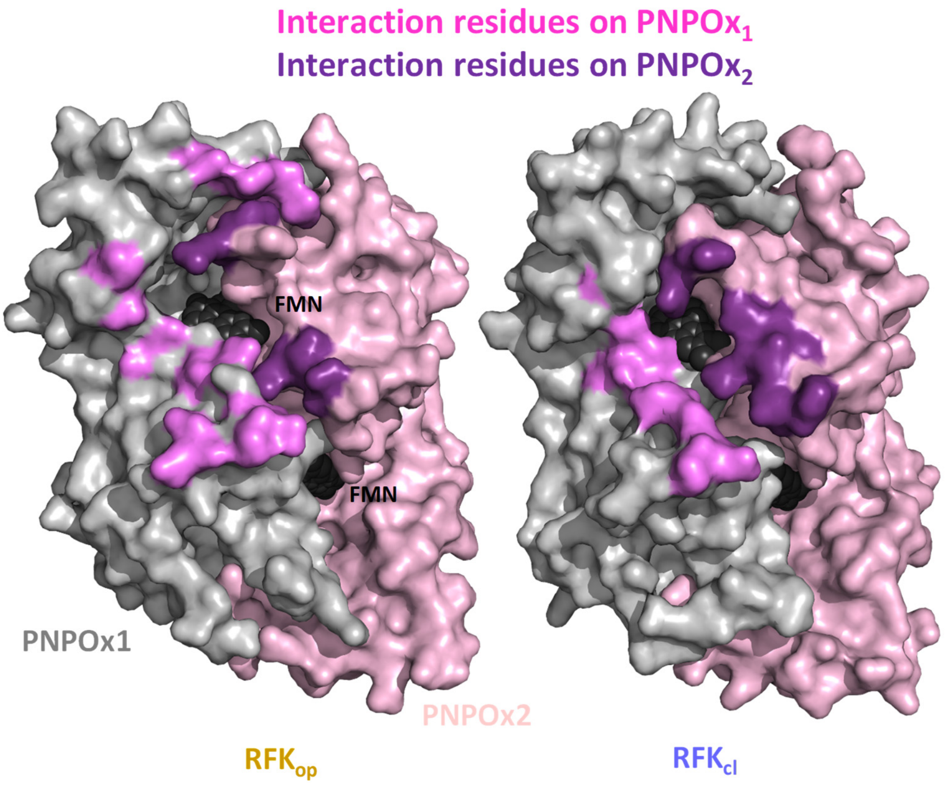

Figure S8. The aPNPOx interaction surface. Hot spots for the interaction on aPNPOx of **(A)** hRFK$_{op}$ and **(B)** hRFK$_{cl}$. The two PNPOx protomers are taken for the final MD simulation structure shown in Figure 7 and respectively coloured in grey and light pink. Hot spots for the interaction of protomers 1 and 2 with RFK are respectively highlighted in pale and dark purple. The two active sites in the PNPOx homodimer are located by the predicted position for the FMN molecule (coming from aligned PDB 1NRG) in black spheres. Both structures for correspond to R2 respectively from Figures S6 and S7.

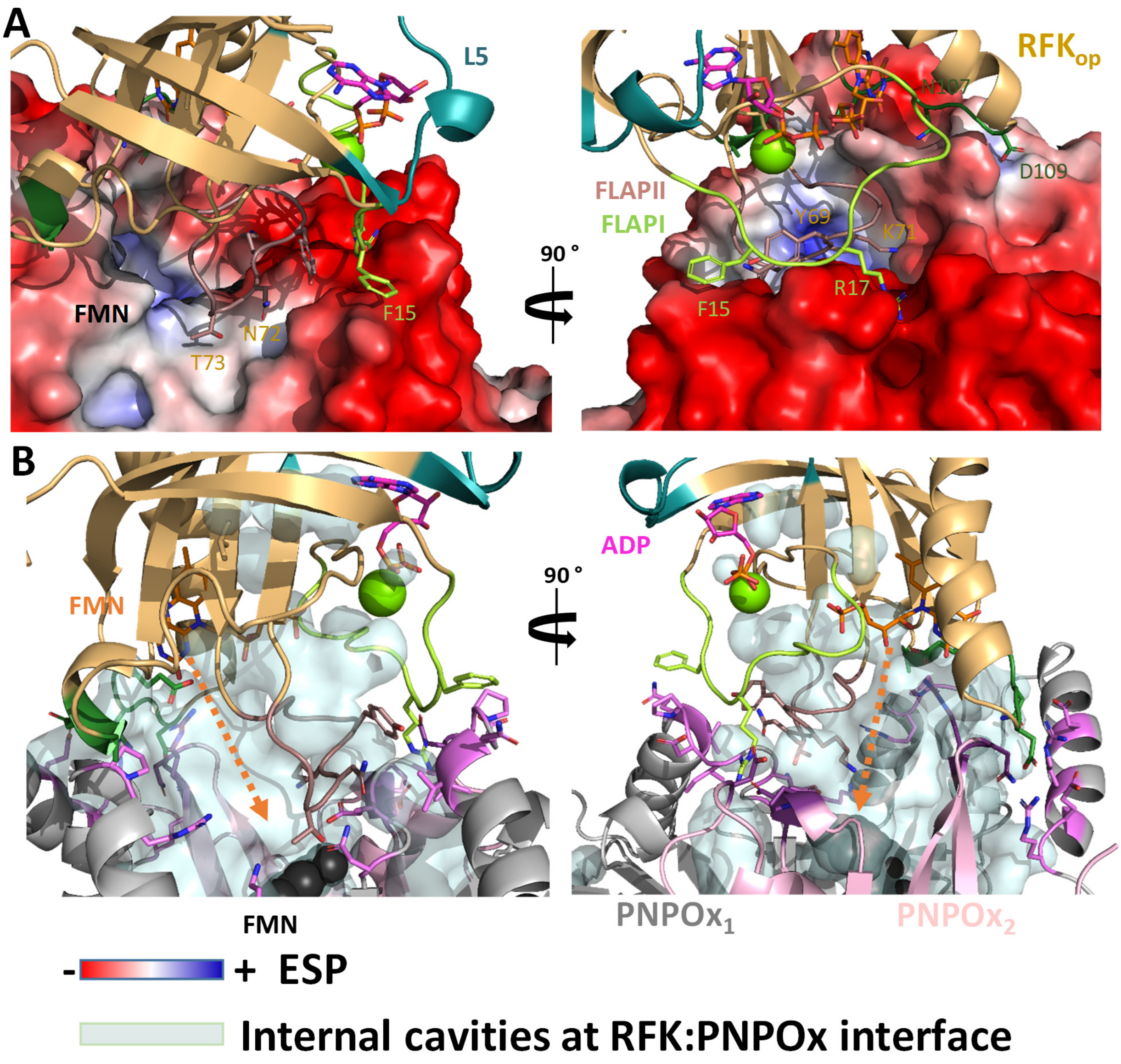

Figure S9. Detail of the molecular coupling of hRFK$_{op}$ to aPNPOx in a representative model (R3). **(A)** Detail of the electrostatic coupling. Interacting residues on hRFK$_{op}$ are shown as CPK coloured sticks, while the aPNPOx homodimer is shown as ESP. **(B)** Internal cavities (transparent pale green) connecting the FMN donor site of RFK and the FMN client site of aPNPOx. Other colour codes as in Figures S7 and S8. Data are shown for R2 from Figure S6.

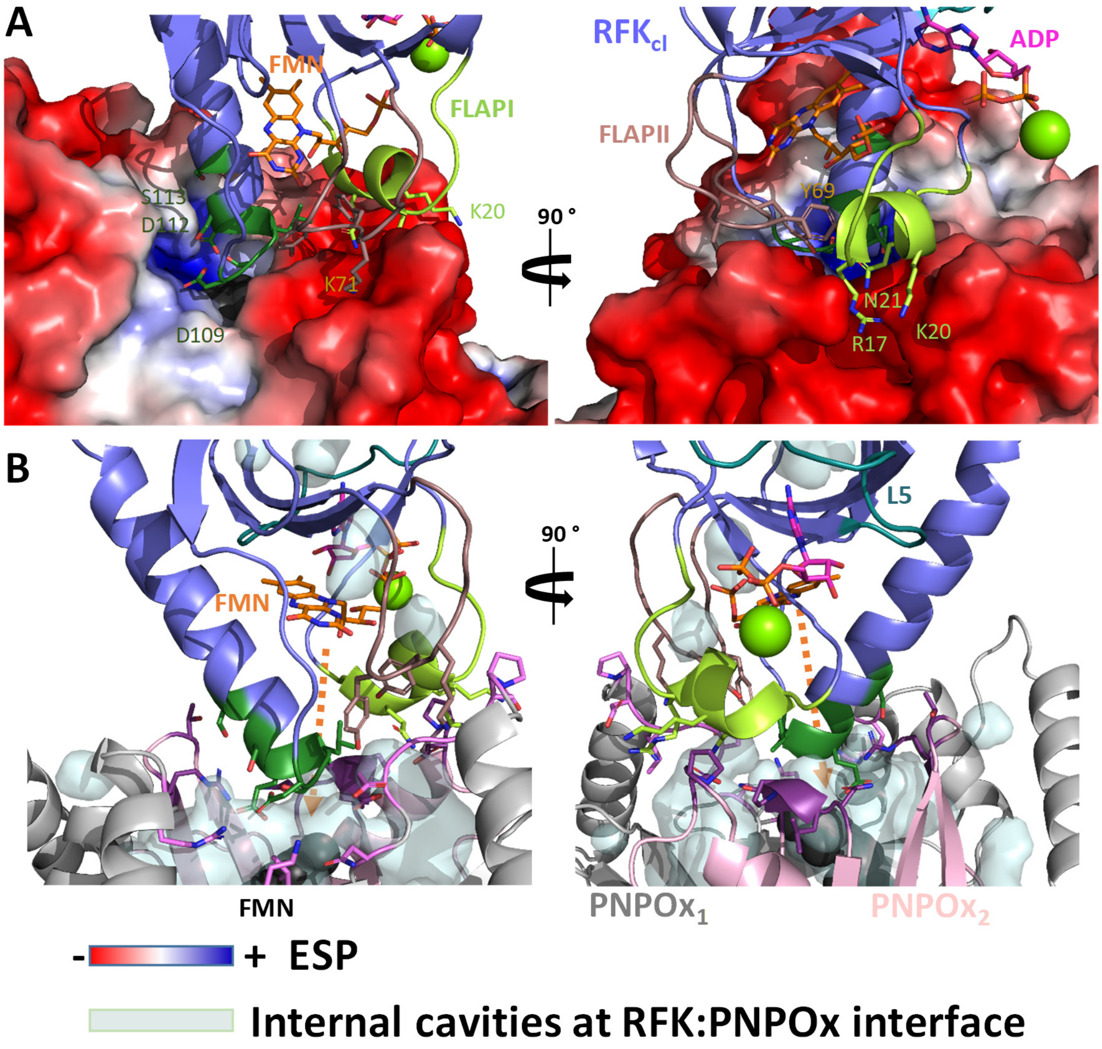

Figure S10. Detail of the molecular coupling of hRFK$_{cl}$ to aPNPOx in a representative model (R3). (A) Detail of the electrostatic coupling. Interacting residues on hRFK$_{cl}$ are shown as CPK coloured sticks, while the aPNPOx homodimer is shown as ESP. **(B)** Internal cavities (transparent pale green) connecting the FMN donor site of RFK and the FMN client site of aPNPOx. Other colour codes as in Figures S7 and S8. Data are shown for R2 from Figure S7.

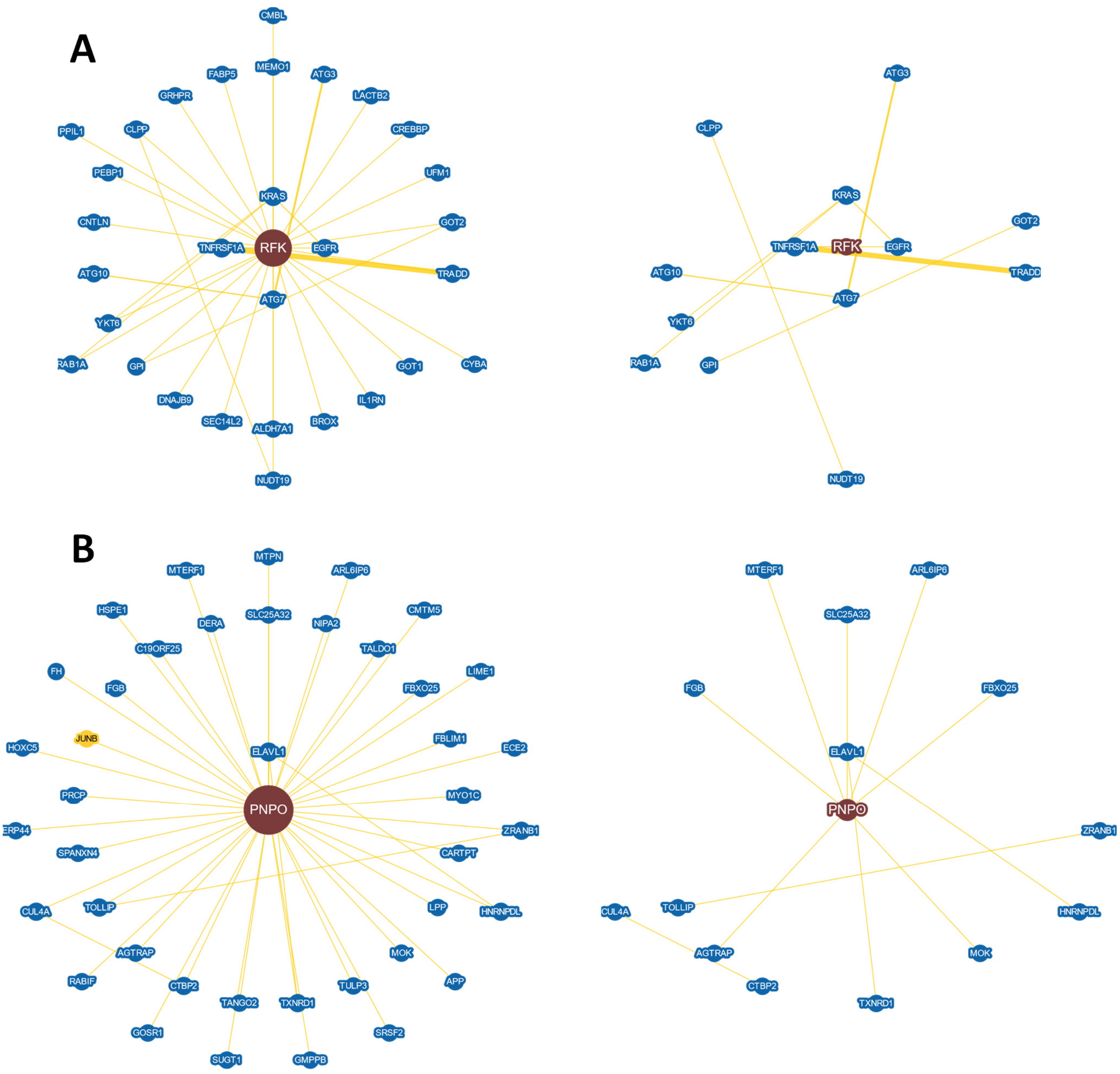

Figure S11. RFK and PNPOx protein:protein interactions networks retrieved from the BioGrid server (https://thebiogrid.org/). Interactions predicted for **(A)** RFK (RFK; Q969G6) and **(B)** PNPOx (PNPO; Q9NVS9). Left and right panels show respectively those interactions with at least one and two independent reports. Data shown are restricted to physical and/or high throughput interactions. Nodes are presented by the alias codes for interactors.

1.2 Supplementary Tables

Table S1. Human FMN-dependent flavoproteins. Flavoproteins were searched according to starting lists provided in (Gudipati et al., 2014) and (Pacheco-Garcia et al., 2021), and updated by new searches in the Uniprot server (https://www.uniprot.org/). Function and catalytic activity was retrieved from the Uniprot server. For sake of simplicity, reactions are indicated only in the forward direction. Protein:protein interactios (PPI) were retrieved from the BioGrid server (https://thebiogrid.org/), and only those with at least two independent reports included number of reports in parenthesis). When available, a representative structure for each flavoprotein is indicated by its PDB code. Data as retrieved by January 2023.

**Name (Gene Name; UniProt code)**	**Function/Catalytic activity (UniProt)**	**PPI (BioGrid)**	**PDB**
Cyanocobalamin reductase / alkylcobalamin dealkylase (MMACHC; Q9Y4U1)	Cobalamin (vitamin B12) cytosolic chaperone that catalyzes the reductive decyanation of cyanocob(III)alamin (cyanocobalamin, CNCbl) to yield cob(II)alamin and cyanide, using **FAD or FMN** as cofactors and NADPH as cosubstrate.<br>2 cob(II)alamin-[cyanocobalamin reductase] + 2 hydrogen cyanide + $NADP^+$ = 2 apo-[cyanocobalamin reductase] + 2 cyanocob(III)alamin + H^+ + NADPH<br>**EC:1.16.1.6**		3SBY
Dihydropyrimidine dehydrogenase (DPYD; Q12882)	Involved in pyrimidine base degradation. Catalyzes the reduction of uracil and thymine. Also involved the degradation of the chemotherapeutic drug 5-fluorouracil.<br>5,6-dihydrouracil + $NADP^+$ $\rightarrow$ H^+ + NADPH + uracil<br>**EC:1.3.1.2**<br>Has both FMN and FAD as cofactors	GOPC (2)	
Dihydroorotate dehydrogenase (DHODH, Q02127)	Catalyzes the conversion of dihydroorotate to orotate with quinone as electron acceptor. Required for UMP biosynthesis via de novo pathway.<br>(*S*)-dihydroorotate + a quinone $\rightarrow$ a quinol + orotate<br>**EC:1.3.5.2**	VAC14 (2), GMPPB (2)	1D3G
FAD synthase (FLAD1; Q8NFF5)	Catalyzes the adenylation of FMN FAD coenzyme.<br>ATP + FMN + H^+ $\rightarrow$ diphosphate + FAD<br>**EC:2.7.7.2**	CDKN1A (2), KLHL14 (2), PLEKHO2 (2), PRTFDC1 (2), REL (2), TRIM23 (2)	
Flavin reductase (NADPH) (BLVRB; P30043)	Broad specificity oxidoreductase that catalyzes the NADPH-dependent reduction of a variety of flavins, such as riboflavin, **FAD or FMN**, biliverdins, methemoglobin and PQQ (pyrroloquinoline quinone). Contributes to heme catabolism and metabolizes linear tetrapyrroles. Can also reduce the complexed Fe^{3+} iron to Fe^{2+} in the presence of FMN and NADPH. In the liver, converts biliverdin to bilirubin.<br>$NADP^+$ + reduced riboflavin $\rightarrow$ 2 H^+ + NADPH + riboflavin	ARL6IP6 (2), FCGR2A (2), FSD1 (2), NENF (2)	1HE4

	**EC:1.5.1.30;** bilirubin IXα + NAD^+ → biliverdin IXα + H^+ + NADH **EC:1.3.1.24;** bilirubin IXα + $NADP^+$ → biliverdin IXα + H^+ + NADPH **EC:1.3.1.24**		
Hydroxyacid oxidase 1 (HAO1; Q9UJM8)	Has 2-hydroxyacid oxidase activity. Most active on the 2-carbon substrate glycolate, but is also active on 2-hydroxy fatty acids, with high activity towards 2-hydroxy palmitate and 2-hydroxy octanoate. (2*S*)-2-hydroxycarboxylate + O_2 → 2-oxocarboxylate + H_2O_2 **EC:1.1.3.15**	CCAR2 (3), KCNAB2 (3)	6GMB
Hydroxyacid oxidase 2 (HAO2; Q9NYQ3)	Catalyzes the oxidation of L-alpha-hydroxy acids as well as, more slowly, that of L-alpha-amino acids. (2*S*)-2-hydroxycarboxylate + O_2 → 2-oxocarboxylate + H_2O_2 **EC:1.1.3.15**		
Iodotyrosine deiodinase 1 (IYD; Q6PHW0)	Catalyzes the oxidative NADPH-dependent deiodination of monoiodotyrosine (L-MIT) or diiodotyrosine (L-DIT). Acts during the hydrolysis of thyroglobulin to liberate iodide, which can then reenter the hormone-producing pathways. Acts more efficiently on monoiodotyrosine than on diiodotyrosine. 2 iodide + L-tyrosine + 2 $NADP^+$ → 3,5-diiodo-L-tyrosine + H^+ + 2 NADPH **EC:1.21.1.1**	DDRGK1 (2) TRIM69 (2)	4TTB
Methionine synthase reductase (MTRR; Q9UBK8)	Key enzyme in methionine and folate homeostasis responsible for the reactivation of methionine synthase (MTR/MS) activity by catalyzing the reductive methylation of MTR-bound cob(II)alamin. Cobalamin (vitamin B12) forms a complex with MTR to serve as an intermediary in methyl transfer reactions that cycles between MTR-bound methylcob(III)alamin and MTR bound-cob(I)alamin forms, and occasional oxidative escape of the cob(I)alamin intermediate during the catalytic cycle leads to the inactive cob(II)alamin species. The processing of cobalamin in the cytosol occurs in a multiprotein complex composed of at least MMACHC, MMADHC, MTRR and MTR which may contribute to shuttle safely and efficiently cobalamin towards MTR in order to produce methionine. Also necessary for the utilization of methyl groups from the folate cycle, thereby affecting transgenerational epigenetic inheritance. Also acts as a molecular chaperone for methionine synthase by stabilizing apoMTR and incorporating methylcob(III)alamin into apoMTR to form the holoenzyme. Also serves as an aquacob(III)alamin reductase by reducing aquacob(III)alamin to cob(II)alamin; this reduction leads to stimulation of the conversion of	MTR (2)	2QTL

	apoMTR and aquacob(III)alamin to MTR holoenzyme.<br>2 [methionine synthase]-methylcob(III)alamin + H^+ + $NADP^+$ + 2 *S*-adenosyl-L-homocysteine → 2 [methionine synthase]-cob(II)alamin + NADPH + 2 *S*-adenosyl-L-methionine<br>**EC:1.16.1.8;**<br>2 cob(II)alamin + H^+ + 2 H_2O + $NADP^+$ → 2 aquacob(III)alamin + NADPH<br>**Has FMN and FAD as cofactors**		
NADPH-cytochrome P450 reductase (POR; P16435)	This enzyme is required for electron transfer from NADP to cytochrome P450 in microsomes. It can also provide electron transfer to heme oxygenase and cytochrome B5.<br>NADPH + 2 oxidized [cytochrome P450] → H^+ + $NADP^+$ + 2 reduced [cytochrome P450]<br>**EC:1.6.2.4**	CYP2E1 (3), FANCC (3), UBXN6 (3), CYP1A2 (2), CYP2C19 (2), CYP2C9 (2)	3QE2
NADH dehydrogenase [ubiquinone] flavoprotein 1, mitocondrial (NDUFV1; P49821)	Core subunit of the mitochondrial membrane respiratory chain NADH dehydrogenase (Complex I) that is believed to belong to the minimal assembly required for catalysis. Complex I functions in the transfer of electrons from NADH to the respiratory chain. The immediate electron acceptor for the enzyme is believed to be ubiquinone.<br>ubiquinone + 5 H^+(in) + NADH = a ubiquinol + 4 H^+(out) + NAD^+<br>**EC:7.1.1.2**	NDUFS3 (6), NDUFA9 (4), NDUFS1 (4), NDUFS2 (4), NDUFA8 (3), NDUFS7 (3), NDUFS8 (3), NDUFV2 (3), C6ORF203 (2), COA3 (2), COX5A (2), CPT1A (2), GRSF1 (2), HSCB (2), ICT1 (2), LONP1 (2), LRPPRC (2), NDUFA2 (2), NDUFS4 (2), NDUFS5 (2), NDUFS6 (2), NDUFV3 (2), PDHA1 (2), PHB2 (2), POR (2), SDHA (2), TOMM40 (2), TUFM (2), VDAC2 (2)	5XTD
NADPH-dependent diflavin oxidoreductase 1 (NDOR1; Q9UHB4)	Component of the cytosolic iron-sulfur (Fe-S) protein assembly (CIA) machinery. Required for the maturation of extramitochondrial Fe-S proteins. Part of an electron transfer chain functioning in an early step of cytosolic Fe-S biogenesis. Transfers electrons from NADPH to the Fe/S cluster of CIAPIN1.<br>NADPH + 2 oxidized [2Fe-2S]-[protein] = H^+ + $NADP^+$ + 2 reduced [2Fe-2S]-[protein]<br>**EC:1.18.1.-**	CIAPIN1 (4), MTUS2 (2), TCF4 (2), TCHP (2)	4H2D

Nitric oxide synthase, brain (NOS1; P29475)	Produces nitric oxide (NO) which is a messenger molecule with diverse functions throughout the body. In the brain and peripheral nervous system, NO displays many properties of a neurotransmitter. Probably has nitrosylase activity and mediates cysteine S-nitrosylation of cytoplasmic target proteins such SRR.<br>$H^+ + 2$ L-arginine + 3 NADPH + 4 $O_2 \rightarrow 4\ H_2O$ + 2 L-citrulline + 3 $NADP^+$ + 2 NO<br>**EC:1.14.13.39**<br>**FMN and FAD as cofactors**	DLG4 (5),<br>PRKD1 (4),<br>STUB1 (4).<br>NOS1AP (3),<br>SOX2 (2),<br>ZDHHC23 (2)	4D1N
Nitric oxide synthase, inducible (NOS2; P35228)	Produces nitric oxide (NO) which is a messenger molecule with diverse functions throughout the body. In macrophages, NO mediates tumoricidal and bactericidal actions. Also has nitrosylase activity and mediates cysteine S-nitrosylation of cytoplasmic target proteins such PTGS2/COX2. As component of the iNOS-S100A8/9 transnitrosylase complex involved in the selective inflammatory stimulus-dependent S-nitrosylation of GAPDH on 'Cys-247' implicated in regulation of the GAIT complex activity and probably multiple targets including ANXA5, EZR, MSN and VIM. Involved in inflammation, enhances the synthesis of proinflammatory mediators such as IL6 and IL8.<br>$H^+ + 2$ L-arginine + 3 NADPH + 4 $O_2 \rightarrow 4\ H_2O$ + 2 L-citrulline + 3 $NADP^+$ + 2 NO<br>**EC:1.14.13.39**<br>**Has FMN and FAD as cofactors**	SPSB2 (9),<br>CALM1 (5),<br>SPSB1 (5),<br>CAV1 (2),<br>CUL5 (2),<br>FBXO45 (2),<br>HSP90AA1 (2),<br>RAC2 (2),<br>SPSB4 (2),<br>UCHL5 (2)	1NSI
Nitric oxide synthase, endotelial (NOS3; P29474)	Produces nitric oxide (NO) which is implicated in vascular smooth muscle relaxation through a cGMP-mediated signal transduction pathway. NO mediates vascular endothelial growth factor (VEGF)-induced angiogenesis in coronary vessels and promotes blood clotting through the activation of platelets.<br>$H^+ + 2$ L-arginine + 3 NADPH + 4 $O_2 \rightarrow 4\ H_2O$ + 2 L-citrulline + 3 $NADP^+$ + 2 NO<br>**EC:1.14.13.39**<br>**FMN and FAD as cofactors**	HSP90AA1 (7),<br>CALM1 (6),<br>CAV1 (4),<br>NOSIP (4),<br>NOSTRIN (4),<br>ACTB (3),<br>AKT1 (3),<br>CDC37 (3),<br>APOE (2),<br>APP (2),<br>GCDH (2),<br>GOLGA2 (2),<br>GUCY1B3 (2),<br>PPP2R4 (2),<br>ST13 (2)	1M9K
Phosphopantotheno ylcysteine decarboxylase (PPCDC; Q96CD2)	Necessary for the biosynthesis of coenzyme A. Catalyzes the decarboxylation of 4-phosphopantothenoylcysteine to form 4'-phosphopantotheine.<br>H^+ + N-[(*R*)-4-phosphopantothenoyl]-L-cysteine $\rightarrow CO_2$ + D-pantetheine 4'-phosphate<br>**EC:4.1.1.36**	ZNF232 (3),<br>FOXR1 (2),<br>TXN2 (2),<br>WDYHV1 (2)	1QZU
Pyridoxine-5'-phosphate oxidase (PNPO; Q9NVS9)	Catalyzes the oxidation of either pyridoxine 5'-phosphate (PNP) or pyridoxamine 5'-phosphate (PMP) into pyridoxal 5'-phosphate (PLP).<br>$H_2O + O_2$ + pyridoxamine 5'-phosphate $\rightarrow H_2O_2$ + NH_4^+ + pyridoxal 5'-phosphate<br>**EC:1.4.3.5**;	AGTRAP (2),<br>ARL6IP6 (3),<br>FBXO25 (3),<br>FGB (2),<br>MOK (3),<br>MTERF1 (3),<br>SLC25A32 (2)	1NRG

	O_2 + pyridoxine 5'-phosphate → H_2O_2 + pyridoxal 5'-phosphate **EC:1.4.3.5**		
Riboflavin kinase (RFK; Q969G6) Biogrid FMN	Catalyzes the phosphorylation of riboflavin (vitamin B2) to form flavin-mononucleotide (FMN), hence rate-limiting enzyme in the synthesis of FAD. Essential for TNF-induced reactive oxygen species (ROS) production. Through its interaction with both TNFRSF1A and CYBA, physically and functionally couples TNFRSF1A to NADPH oxidase. TNF-activation of RFK may enhance the incorporation of FAD in NADPH oxidase, a critical step for the assembly and activation of NADPH oxidase. ATP + riboflavin → ADP + FMN + H^+ **EC:2.7.1.26**	TNFRSF1A (2)	
Squalene monooxygenase (SQLE; Q14534)	Catalyzes the stereospecific oxidation of squalene to (S)-2,3-epoxysqualene, and is considered to be a rate-limiting enzyme in steroid biosynthesis. O_2 + reduced [NADPH—hemoprotein reductase] + squalene → (*S*)-2,3-epoxysqualene + H^+ + H_2O + oxidized [NADPH—hemoprotein reductase] **EC:1.14.14.17**	FAF2 (2), MARCH6 (2), TREML2 (2)	6C6N
tRNA-dihydrouridine(16/17) synthase [NAD(P)(+)]-like (DUS1L; Q6P1R4)	Catalyzes the synthesis of dihydrouridine, a modified base found in the D-loop of most tRNAs. 5,6-dihydrouridine$_{16}$ in tRNA + $NADP^+$ = H^+ + NADPH + uridine$_{16}$ in tRNA. **EC:1.3.1.88**	HJURP (2)	
tRNA-dihydrouridine(20) synthase [NAD(P)$^+$]-like (DUS2L; Q9NX74)	Catalyzes the NADPH-dependent synthesis of dihydrouridine, a modified base found in the D-loop of most tRNAs. Negatively regulates the activation of EIF2AK2/PKR. 5,6-dihydrouridine^{20} in tRNA + $NADP^+$ → H^+ + NADPH + uridine^{20} in tRNA **EC:1.3.1.91**	EIF2AK2 (3), PRKRA (3), KANSL1 (2)	4XP7
tRNA-dihydrouridine(47) synthase [NAD(P)(+)]-like (DUS3L; Q96G46)	Catalyzes the synthesis of dihydrouridine, a modified base, in various RNAs, such as tRNAs, mRNAs and some long non-coding RNAs (lncRNAs). Mainly modifies the uridine in position 47 (U47) in the D-loop of most cytoplasmic tRNAs. 5,6-dihydrouridine$_{47}$ in tRNA + NAD^+ = H^+ + NADH + uridine$_{47}$ in tRNA This reaction proceeds in the backward direction. **EC:1.3.1.89**		
tRNA-dihydrouridine(20a/20b) synthase [NAD(P)+]-like (DUS4L; O95620)	Catalyzes the synthesis of dihydrouridine, a modified base found in the D-loop of most tRNAs. 5,6-dihydrouridine(20a) in tRNA + $NADP^+$ = H^+ + NADPH + uridine(20a) in tRNA This reaction proceeds in the backward direction. **EC:1.3.1.90**	GCA (2), PTPRG (2)	

REFERENCES

GUDIPATI, V., KOCH, K., LIENHART, W. D. & MACHEROUX, P. 2014. The flavoproteome of the yeast Saccharomyces cerevisiae. *Biochim Biophys Acta,* 1844**,** 535-44.

PACHECO-GARCIA, J. L., ANOZ-CARBONELL, E., VANKOVA, P., KANNAN, A., PALOMINO-MORALES, R., MESA-TORRES, N., SALIDO, E., MAN, P., MEDINA, M., NAGANATHAN, A. N. & PEY, A. L. 2021. Structural basis of the pleiotropic and specific phenotypic consequences of missense mutations in the multifunctional NAD(P)H:quinone oxidoreductase 1 and their pharmacological rescue. *Redox Biol,* 46**,** 102112.
